# Genetic variation at hair length candidate genes in elephants and the extinct woolly mammoth

**DOI:** 10.1186/1471-2148-9-232

**Published:** 2009-09-11

**Authors:** Alfred L Roca, Yasuko Ishida, Nikolas Nikolaidis, Sergios-Orestis Kolokotronis, Stephen Fratpietro, Kristin Stewardson, Shannon Hensley, Michele Tisdale, Gennady Boeskorov, Alex D Greenwood

**Affiliations:** 1 Department of Animal Sciences, University of Illinois at Urbana-Champaign, Urbana, IL 61801, USA; 2 Department of Biological Science, College of Natural Sciences and Mathematics, California State University at Fullerton, Fullerton, CA 92834, USA; 3 Sackler Institute for Comparative Genomics, American Museum of Natural History, New York, NY 10024, USA; 4 Paleo-DNA Laboratory, Lakehead University, Thunder Bay, ON P7B 5E1, Canada; 5 Department of Biological Sciences, Old Dominion University, Norfolk, VA 23529, USA; 6 Institute for Diamond and Precious Metals Geology, Siberian Branch of Russian Academy of Sciences, Yakutsk, Russian Federation

## Abstract

**Background:**

Like humans, the living elephants are unusual among mammals in being sparsely covered with hair. Relative to extant elephants, the extinct woolly mammoth, *Mammuthus primigenius*, had a dense hair cover and extremely long hair, which likely were adaptations to its subarctic habitat. The fibroblast growth factor 5 (*FGF5*) gene affects hair length in a diverse set of mammalian species. Mutations in *FGF5 *lead to recessive long hair phenotypes in mice, dogs, and cats; and the gene has been implicated in hair length variation in rabbits. Thus, *FGF5 *represents a leading candidate gene for the phenotypic differences in hair length notable between extant elephants and the woolly mammoth. We therefore sequenced the three exons (except for the 3' UTR) and a portion of the promoter of *FGF5 *from the living elephantid species (Asian, African savanna and African forest elephants) and, using protocols for ancient DNA, from a woolly mammoth.

**Results:**

Between the extant elephants and the mammoth, two single base substitutions were observed in *FGF5*, neither of which alters the amino acid sequence. Modeling of the protein structure suggests that the elephantid proteins fold similarly to the human FGF5 protein. Bioinformatics analyses and DNA sequencing of another locus that has been implicated in hair cover in humans, type I hair keratin pseudogene (*KRTHAP1*), also yielded negative results. Interestingly, *KRTHAP1 *is a pseudogene in elephantids as in humans (although fully functional in non-human primates).

**Conclusion:**

The data suggest that the coding sequence of the *FGF5 *gene is not the critical determinant of hair length differences among elephantids. The results are discussed in the context of hairlessness among mammals and in terms of the potential impact of large body size, subarctic conditions, and an aquatic ancestor on hair cover in the Proboscidea.

## Background

Hair is a defining characteristic of mammals. The hair follicle is the only organ in mammals to undergo life-long cycles of growth, regression and quiescence [1]. Hair development proceeds through a cycle of anagen in which hair follicles undergo rapid growth, catagen in which hair growth ceases due to apoptosis-driven regression, and telogen in which the hair follicle enters a period of relative quiescence [1]. Longer hair can thus result from an increase in the length of time during which anagen proceeds. A loss-of-function mutation in the fibroblast growth factor 5 gene (designated *Fgf5 *in mice and rats, and *FGF5 *in other mammals) is responsible for the long hair phenotype present in angora mice, while a similar long hair phenotype occurs in mice homozygous for a null allele of *Fgf5 *produced by gene targeting [2]. In mice, catagen does eventually occur even in the absence of functional FGF5, indicating that other factors are also involved in the cycle [2].

**Figure 1:**
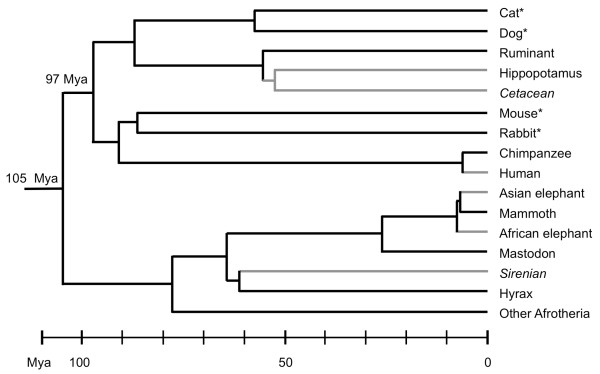
**Chronogram showing divergence dates for selected mammalian species**. Branches are shown in gray for relatively hairless lineages [28] and in black for taxa with greater hair cover. Lineages that are completely aquatic are italicized. An asterisk indicates domestic species for which mutations in the *FGF5 *gene have been identified as responsible for long hair phenotypes in one or more breeds [2,5,6,8]. The relationships depicted among taxa, and the divergence dates on the chronogram, are from previously published paleontological [30,31] or genetic [9,10,34,39] studies.

The *FGF5 *gene consists of three exons [3]. In wild-type mice and other mammals, the *FGF5 *transcript is present in two isoforms, with the smaller transcript due to alternative splicing in which exon 2 is excluded from the mRNA [3,4]. The shorter transcript antagonizes the activity of the longer transcript, suggesting that they function together in hair cycle regulation [4]. In mice, the *Fgf5 *mutation causing the long-hair angora phenotype affects exon 1 [2]. In dogs, sequencing of the *FGF5 *gene in 218 individuals from 14 breeds, including three dog breeds fixed for long hair and five breeds fixed for short hair, identified a missense mutation in exon 1 as responsible for the long haired phenotype [5]. In domestic cats, the *FGF5 *gene was shown to be associated with hair length [6,7], with four independent mutations in exon 1 or 3 considered to be functionally significant in controlling hair length in a survey of more than 380 individuals from 26 short- or long-haired breeds, non-breed cats and two pedigrees [6]. In rabbits, mutations in *FGF5 *have been reported to have significant association with wool yield [8]. Given that the species for which *FGF5 *is known to influence hair length belong to different superordinal placental clades that diverged ca. 97 million years ago (Mya) (Figure 1) [9,10], it seems plausible to hypothesize that *FGF5 *may be a critical determinant of hair length across mammals.

The Elephantidae, a family of proboscideans comprising the living elephants, their extinct relatives, and the extinct mammoths [11], would constitute an important group for the comparative study of genes involved in regulating hair cover and growth. The woolly mammoth, *Mammuthus primigenius*, was covered with hair ranging in length from a few centimeters to over 90 cm, with coarse outer hairs, beneath which were shorter, thinner hairs forming densely packed underwool (maximum 2.5-8 cm long) that formed a thermal insulating layer [12,13]. By contrast, extant elephants are sparsely covered with hair and have hair of short length [12]. Given the role of *FGF5 *in determining hair length in a diverse set of mammalian taxa, we hypothesized that loss of function of this gene may play a role in the longer hair length of the woolly mammoth. We therefore generated and compared sequences of *FGF5 *from living elephants and the woolly mammoth. In addition, using a combination of bioinformatics analysis and DNA sequencing, another gene related to hair phenotype in humans, type I hair keratin pseudogene (*KRTHAP1*) [14], was examined, as were three other genes coding for hair keratin proteins [15].

## Results

The *FGF5 *gene was sequenced from a woolly mammoth and from two Asian elephants (*Elephas maximus*), two African savanna elephants (*Loxodonta africana*) and two African forest elephants (*L. cyclotis*) (Table 1) [16]. For the woolly mammoth, protocols established for ancient DNA were used [17], with the complete *FGF5 *coding sequence obtained for Indigirka mammoth N2031, a ca. 11000-13000 year-old tooth from the Indigirka River basin, Federal Republic of Russia. PCR products from the Indigirka mammoth were amplified from multiple extracts at two different laboratories (Norfolk and Thunder Bay), with 2 or more PCRs performed per fragment, and PCR fragments cloned and sequenced. Among-clone variation was observed but no consistent differences among amplifications were detected. Partial sequence was also obtained from the Jarkov mammoth, a ca. 20,380 year-old sample from the Taimyr Peninsula, Federal Republic of Russia. (See Methods and Additional file 1 for further information on the mammoths and laboratory protocols and primers).

**Table 1:** Alignment of variable sites among elephantids for the *FGF5 *gene

		**Promoter**								**5' UTR**		**ORFs**					
										
										**Exon 1**					**Exon 2**	**Exon 3**	
		
**Species**	**Sample no**.	**-314**	**-290**	**-269**	**-265**	**-150**	**-112**	**-76**	**-1**	**68**	**80**	**189**	**327**	**427**	**Identical**	**757**	**790**
*E. maximus*	Ema-10	G	C	G	G	C	G	C	A	C	C	T	G	A		G	G
*E. maximus*	Ema-6	.	.	.	.	.	.	.	.	.	.	T/C	.	A/G	-	C	.

*L. cyclotis*	Lcy-LO3508	.	T	C	.	G	C	.	.	.	G	C	C	G	-	C	.
*L. cyclotis*	Lcy-LO3505	.	T	C	T	G	C	.	A/:	.	G	.	C	G		C	.
*L. africana*	Laf-KR0014	A	.	C	.	G	C	.	.	T	.	.	.	G		C	.
*L. africana*	Laf-KR0138	A	.	C	.	G	C	.	.	T	.	.	.	G	-	C	.

*M. primigenius*	Indigirka	-	.	C	.	G	C	**T**	.	.	.	.	.	G		C	**A**

Asian elephant Ema-10 is used as the reference sequence. Dots represent identity to the reference. Differences are shown as the base (both bases if the site was heterozygous). The A/: at -1 for Lcy-LO3505 indicates a site with a (heterozygous) single nucleotide deletion. Sites with character states unique to the woolly mammoth are in boldface. Variable sites are numbered from the putative start position of exon 1. Exon 2 was identical across species. Dash indicates sequences not generated for a sample.

The complete 5' UTR, complete open reading frames (ORFs) of the three exons, and part of the promoter region of the *FGF5 *gene were sequenced in the elephants and the Indigirka woolly mammoth (Table 1). All of the elephants as well as the woolly mammoth had an uninterrupted open reading frame (without premature stop codons). In each of the elephantids, exon 1 was 593 bp in length (229 bp of 5' UTR, 364 bp of coding region); exon 2 was 104 bp; while the protein coding region of exon 3 was 348 bp, with 40 bp of the 3' UTR also sequenced. The four boundaries between exons and introns were identical across all elephantids sequenced, thus *FGF5 *does not vary at these splice sites among elephantids. Full sequencing of the two introns was not attempted due to their length: 7,729 bp and 11,312 bp for introns 1 and 2, respectively, in human, with even longer introns present in savanna elephant based on genomic traces (data not shown). Additionally, the mammoth genomic sequences [15] were found to have poor coverage of both introns in the mammoth (data not shown).

Only two mammoth-specific differences were found: one in the promoter and one in exon 3 (Table 1). The substitution in the promoter sequence (Table 1) did not alter any predicted transcription factor binding sites [18]. The difference present in exon 3 of the mammoth was a guanine to adenine substitution at position 790 that was a silent mutation, i.e. did not alter the amino acid sequence (Table 1). This substitution also did not lead to a rare codon being present in the mammoth. The mammoth FGF5 amino acid sequence was identical to that of 2 of the 3 extant elephant species. This suggested that all elephantids including the long-haired mammoths had a functional FGF5 protein.

**Figure 2:**
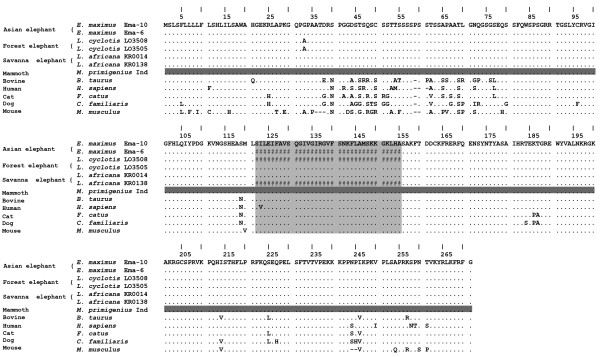
**Alignment of FGF5 amino acid sequences determined for elephantids, along with the large splice variants of bovid, human, cat, dog (wolf, not shown, has amino acid sequence identical to dog), and mouse**. Exon 2 is lightly shaded while exons 1 and 3 are unshaded. Common and scientific names are shown for all species; laboratory codes are shown for the elephantids (see Methods for information on individual samples). An Asian elephant is used as the reference sequence; identities are shown as dots; differences are shown as the single letter amino acid code that differs from the reference sequence; alignment gaps are shown as dashes. The Indigirka ("Ind") woolly mammoth sequence is distinguished by dark shading. Sequences not obtained for specific individuals are shown with #.

**Figure 3:**
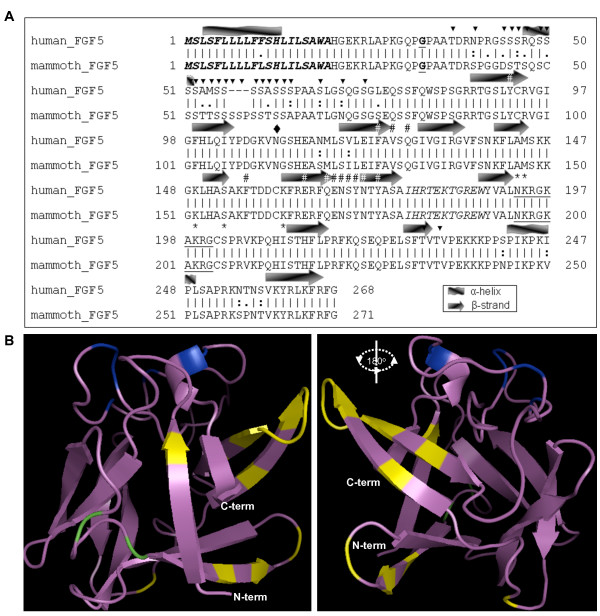
**The mammoth FGF5 protein compared to the human FGF5 protein sequence**. (A) Pairwise alignment of the FGF5 sequences from human [GenBank:NP_004455] and mammoth (this study). The secondary structure, which is shown above each alignment row, represents the consensus structure as predicted by the SSPro and PHYRE programs. The signal peptide is shown with bold-italic fonts; the position of the G33A forest elephant mutation is depicted with bold-underlined fonts; the solvent exposed loop is shown with italics; the glycine box is shown with underlined font [23]. Black triangles depict O-glycosylation sites and a black diamond is used to depict the N-glycosylation site. The FGF receptor (FGFR) binding sites are shown with # and the heparin binding sites with * [24]. (B) The three-dimensional model of the mammoth FGF5 (violet color) protein. The FGFR and heparin binding sites are depicted using yellow and blue color, respectively. The differences between human and mammoth FGF5 sequences are colored green.

The nucleotide sequence of *FGF5 *varied among living elephantids (Table 1), although only one non-synonymous substitution was found among living or extinct elephantids. In both forest elephant individuals, exon 1 (nucleotide position 327) contained a codon for glycine at residue 33, whereas a codon for alanine was present in other elephantids at this position (Table 1, Figure 2). This is a physicochemically conservative change [19]. Among other mammals for which *FGF5 *sequence is available, this G33A mutation was found to be present only in the in rodent FGF5 protein sequence (Figure 2). The mutation is located at the N-terminus of the protein (Figure 2). The N- and C-termini of other members of the FGF family play key roles in the specificity of interaction with the FGF-receptors (FGFRs). [20]. However, this mutation resides within a mainly unstructured region, predicted to be an extended loop downstream of the signal peptide (Figure 3A and data not shown). Thus, the G33A mutation in forest elephants would be unlikely to affect the secondary structure of the FGF5 protein. Both glycine and alanine are nonpolar, neutral amino acids, with neighboring hydropathy indices [21,22] in the hydrophobic range, which precludes prediction of their structural position, i.e. external or internal. The tertiary structure of this region could not be modeled, because the structure of this region has not been experimentally resolved in any member of the FGF family. Analysis using SIFT and POLYPHEN programs suggested that while the G33A mutation may increase the stability of the protein, the G33A mutation in forest elephants should not have serious consequences on FGF5 protein function (data not shown).

Human and mammoth FGF5 protein sequences were also compared [23,24]. FGF5 is predicted to be secreted and has an almost identical signal peptide sequence for the two species (Figure 3A). The major difference between the two sequences is an insertion/deletion of three amino acids at the N-terminal region. This region is predicted to include many O-glucosylation sites, suggesting a putative difference in glycosylation between the two FGF5 proteins (Figure 3A). The two proteins are predicted to have a unique N-glycosylation site at position 110 of the human sequence.

The general features of the amino acids of mammalian FGF5 are shown in Additional file 1, as are the phylogenetic relationships among FGF5 amino acid sequences. The FGF5 proteins are very similar across elephantids but differ from those of other mammalian FGF5 proteins. To test whether any of these differences would be predicted to alter the three-dimensional structure of the elephantid FGF5 proteins, the tertiary structures of FGF5 in different species were predicted. This analysis predicted that all described mammalian FGF5 proteins fold similarly (data not shown). The structural analysis also revealed that the amino acid differences between human and mammoth FGF5 sequences (shown in green color in Figure 3B) do not correspond to residues known to interact with the FGF receptor (FGFR, yellow color) and heparin (blue color) (Figure 3B). The two amino acid differences between humans and mammoths that are included in the 3D model are predicted to be parts of loops and do not seem to affect the secondary or the tertiary conformation of the FGF5 molecule (Figure 3B).

To examine the quality of sequence traces for the recently published genome sequence of the woolly mammoth [15], the mammoth *FGF5 *DNA sequences generated for this study were compared using BLAST to homologous sequences generated by the Mammoth Genome Project [15]. Five matching mammoth genome sequences were found, comprising sequence coverage of about 50% (714/1434 bp). Coverage of the mammoth genome varied by region for the *FGF5 *gene. Four genomic sequences matched the promoter and 5' UTR; these covered 99% (513/520 bp) of the corresponding region sequenced by the current study. There were 6 discrepancies among the traces covering this region. Only one genomic sequence was found that overlapped with the coding regions, and it covered only 22% (201/915 bp) of the sequence determined for the current study, with nine discrepancies found between genomic traces and our sequences. Overall, four of the five mammoth genomic sequences had discrepancies with the mammoth sequences generated for the current study, with a total of 15 nucleotide site discrepancies detected. The discrepancies relative to our sequence likely reflect damage present in the ancient DNA of the mammoths used to generate genomic sequences. Similar ancient DNA damage affected the mammoth sequences generated for the current study (although for the current study multiple clones from at least two independent PCRs per fragment were used to successfully generate a consensus sequence). For five PCR amplicons used in the current study to determine the sequence of the *FGF5 *promoter, the among-clone diversity was in the range 0-9 (see Additional file 1). Unlike PCR-based approaches where multiple PCRs can be performed and multiple clonal sequences per PCR determined to generate a consensus sequence, the mammoth genome does not currently have high enough coverage per base to be confident that observed differences among traces, individuals or species represent the true sequence rather than ancient DNA damage. Thus although the mammoth genome is extremely useful for designing mammoth-specific primers and for initial queries, our data suggest that PCR, cloning and sequencing would still be required to determine mammoth DNA sequences, to account both for ancient DNA damage and gaps in the low-coverage genome sequences.

**Figure 4:**
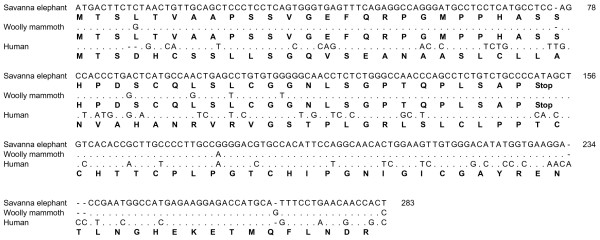
**Partial sequence of *KRTHAP1 *exon 1 of the Indigirka woolly mammoth (*Mammuthus primigenius*), aligned to respective sequences from savanna elephant (*Loxodonta africana*) and human (*Homo sapiens*)**. For each species, both DNA (above) and amino acid sequences (below, in boldface) are shown. Premature stop codons predicted for the elephantids are indicated (after which no amino acids are shown for them). Mammoth consensus sequence was generated from clones of two independent PCR reactions (not shown); genomic sequences were used for the other two species.

Like *FGF5*, other loci have been identified that are associated with reduced hair cover. In humans the type I hair keratin pseudogene *KRTHAP1 *has a premature stop codon in the fourth exon, and protein is not detected in human hair follicles [14]. In great apes, the orthologous gene has an intact ORF, with RNA expressed and protein translated in the hair follicles of chimpanzees (*cHaA*) and gorillas (*gHaA*) [14]. Thus, while closely related primates with dense hair coverage express this gene, relatively hairless humans do not. Using the *Loxodonta africana *draft genome sequence, all of the homologous exons except for exon 7 for this gene were identified. Exon 1 displayed a predicted premature stop codon (Figure 4). Thus, as in humans, this gene appeared to be disrupted in the savanna African elephant. A 302 bp segment of exon 1 was therefore amplified and sequenced from the Indigirka mammoth to examine the region that contained the premature stop codon in the elephant. The stop codon was found to be present in the mammoth as well (Figure 4), suggesting that this mutation is not involved in hair phenotype differences among elephantids.

Among mammals, hair phenotype is affected by hair keratin genes [25]. We therefore examined keratin genes reported as displaying either elephant or mammoth unique differences [15], using genomic sequences of savanna elephant or woolly mammoth [15,26]. *KRT25 *was identified as having a unique alanine to serine change, but this was specific to only one of two woolly mammoths previously sequenced [15]. Similarly, in the elephantids *KRT27 *and *KRT83 *were found to code for rare amino acid differences. However, only a methionine to valine change in KRT27 was found to be unique to mammoths, while the methionine present in elephant KRT27 was also present in the fully hair-covered hyrax. Thus the differences in *KRT27 *and *KRT83 *are unlikely to be associated with differences in hair cover.

## Discussion

To date, the *FGF5 *mutations found to produce long hair have been uncovered in phenotypic variants among laboratory or domestic mammals, including mice, rabbits, dogs and cats (Figure 1) [2,5,6,8]. A role for *FGF5 *in inter- as opposed to intra-species differences in hair length has not been established. Nonetheless, the association of *FGF5 *mutations with long-hair phenotypes in a wide variety of distantly related mammals (Figure 1) suggested that *FGF5 *might be a determinant of hair length in mammals in general. To test this hypothesis we sequenced the open reading frames of all three exons, the 5' UTR and the promoter region of the *FGF5 *gene in the relatively hairless extant elephantids and in the woolly mammoth. Our data show that these regions of *FGF5 *are highly conserved among elephantids, including the woolly mammoth. Only one variant in the amino acid sequence was detected among elephantids, the G33A mutation in forest elephants coded by exon 1. However, our analysis suggested that this mutation would not greatly affect protein function, a conclusion also supported by the presence of same amino acid substitution in the wild-type sequence of FGF5 in murid rodents. While regulatory mechanisms may exist that would not be detectable by our study, and a role for *FGF5 *in the long hair of mammoths cannot be completely ruled out, the most parsimonious interpretation of our results suggests that *FGF5 *was not the major genetic determinant of long hair in mammoth.

Similarly, no differences were found among elephantids for partial sequences of several additional candidate genes such as *KRTHAP1*, *KRT25*, *KRT27*, and *KRT83*. Thus, none of the candidate genes examined thus far demonstrated a clear difference exclusive to mammoths, which would be necessary for establishing a role in their unique dense and long-haired phenotype relative to extant elephants. While a host of additional genes are known to influence hair development, many play other critical developmental roles and would likely be lethal if function were perturbed [25]. Thus, future candidate genes will likely reside among the keratin and keratin-associated protein (*KRTAP*) genes, believed to play a role in the evolution of mammalian hair characteristics [27]. Among mammals, *KRTAP *gene repertoires vary considerably, with homogenization within groups [27], although the genes have not been catalogued in elephants or other afrotheres. Once the savanna elephant genome is complete, keratin and *KRTAP *genes from this species may be identified as candidates for determining the hair differences among elephantids.

Among living mammals hairlessness is more pronounced among fully aquatic species of sirenians and cetaceans (Figure 1); thus the designation of humans and elephants as "hairless" is a relative term [28]. In the case of elephants, hairlessness may be a thermoregulatory adaptation to large body size [28], which would be consistent with a gain of hair cover for the woolly mammoth [12,29], since mammoths appear first in Africa before the lineage adapted to colder environments [11]. In considering the evolution and genetics of hair cover in extant elephants, woolly mammoths and other proboscideans, a number of factors must be taken into account. First, it is difficult to determine based on outgroups whether hair cover was lost in extant elephantids or gained in the woolly mammoth. The presence of considerable hair cover in a distantly related outgroup to the elephantids, the American mastodon (*Mammut americanum*) [30,31], does not necessarily suggest that hair cover is ancestral. Hair cover in the American mastodon may comprise a convergent adaptation to cold and/or aquatic habitats, rather than an ancestral state [12]. Second, the proboscidean lineage that gave rise to both elephantids and mastodons is likely to have derived from aquatic or semi-aquatic ancestors [32,33]. Although many semi-aquatic species are not hairless [28], proboscideans derive from a common ancestor with the fully aquatic and hairless sirenians [32]; while Proboscidea and Sirenia, along with Hyracoidea (hyraxes, which are not hairless), comprise the Paenungulata, one of the few unresolved trichotomies among extant mammalian orders [9]. Additionally, some ancestral proboscideans were as large as living elephants [32], and if hairlessness is an adaptation to large body size in terrestrial mammals, it may have been the ancestral state in proboscideans [12,28,29].

A third consideration is that, both in the case of the hyrax-sirenian-proboscidean clade and the elephantid clade, the evidence suggests that divergence of the ancestral line into two and then three descendent lineages occurred in quick succession. Among the elephantids, nearly complete mtDNA sequences have been generated for all three genera including mammoths [34-36]; using the mastodon mito-genome as an outgroup suggests that *Loxodonta *diverged from the common ancestor of *Elephas *and *Mammuthus *ca. 7.6 Mya; followed by the divergence of the two latter genera ca. 6.7 Mya (Figure 1) [36]. The order of divergence among the Paenungulata remains unresolved [9], suggesting, as in the case of the elephantids, that the two divergences that yielded the three mammalian orders occurred in rapid succession. The rapid divergence of lineages in both cases suggests that incongruent lineage sorting of alleles may have affected many loci, causing discrepancies between gene and species trees [37-39]. Interestingly, the mammoth and African elephant *FGF5 *sequences are identical at positions -112, -150 and -269 of the promoter (Table 1), while differing from the Asian elephant sequence even though the Asian elephant and mammoth are sister taxa [36]. This suggests that this region of the genome may have been subject to incongruent lineage sorting in which the gene tree does not match the species tree [37], as has been reported for other gene segments [38]. Thus both convergent evolution and incongruent lineage sorting may have affected genes involved in hair cover among the Proboscidea.

## Conclusion

Although the gene for long hair in mammoths was not here identified, proboscideans remain an important group for understanding the evolution of hair cover. While most mammals have dense hair cover, humans and extant elephants are notable in being relatively hairless [28], and both are closely related to species with much greater hair cover (great apes and woolly mammoths, respectively). Both lineages are also noteworthy in being "genome-enabled" [40] for the study of genes affecting hair cover. The human and chimpanzee genomes have been sequenced [41], while the elephant genome is being sequenced [26], and substantial coverage for the mammoth genome is now available [15]. Other than aquatic species, the number of other mammalian genera considered to be "hairless" is quite small [28]. Thus, for a comparative approach to the evolution of hair cover, proboscideans comprise an important group for further research.

## Methods

### Samples

Modern elephant DNA was extracted from blood or tissue samples. Wild African savanna elephants Laf-KR0014 and Laf-KR0138 were from Kruger National Park, South Africa. Wild African forest elephants Lcy-LO3505 and Lcy-LO3508 were from Lopé National Park in Gabon. Asian elephants Ema-6 and Ema-10 were zoo animals at the Rosamond Gifford Zoo at Burnet Park, Syracuse, NY. Both Ema-6 (North American studbook number 27) and Ema-10 (North American studbook number 28) had been wild-caught, most likely in Thailand.

The mammoth tooth designated N2031, which is the focus of this project, is from the Indigirka River basin, Russian Federation. N2031 was found in 1965 on the Berelekh river (a tributary of the Indigirka river), in the Berelekh mammoth "cemetery" *in situ*. The approximate geological age is 11000 - 13000 years before present (BP; G. Boeskorov, personal communication). The sample was originally obtained from the Geological Museum, Geological Institute, Yakutsk. Partial sequences were also obtained from the Jarkov mammoth discovered in the Taimyr Pensinsula, Russian Federation and dated to ca. 20,380 years BP [42]. In order to obtain material for DNA extraction, an electric drill with individual sterile drill bits were used at low speed to collect the bone powder and shavings.

### DNA extraction, PCR, and sequencing

Extractions of mammoth samples in Norfolk were carried out in a room dedicated to ancient DNA work in a CleanSpot PCR hood (Coy Laboratory) following an established protocol [43]. Likewise, all pre-amplification work in Thunder Bay was performed in a 'Clean Lab'. PCR amplifications were performed at least twice per primer pair. Primer sequences and details of ancient DNA extractions, PCR and sequencing are included in Additional file 1. All PCR products were cloned and sequenced since direct sequencing can lead to an erroneous sequence due to contamination and DNA damage in the extract. Cloning and sub-sampling individual representative amplified sequences provides a better representation of the original template amplified [44], therefore none of the mammoth consensus sequences generated in this study were determined from direct sequencing.

DNA from extant elephants (~50 ng) underwent amplification by PCR using 200 nM final concentration of each oligonucleotide primer in 1.5 mM MgCl_2_, with AmpliTaq Gold DNA Polymerase (Applied Biosystems Inc. [ABI]). Primers are listed in Additional file 1. For all primer pairs, PCR consisted of an initial 95°C for 9:45 min; with cycles of 20 sec at 94°C, followed by 30 sec at 60°C (3 cycles); 58°C, 56°C, 54°C, or 52°C (5 cycles each temperature); or 50°C (last 22 cycles), followed by 30 sec extension at 72°C; with a final extension of 3 min at 72°C. PCR products were enzyme-purified [45] and sequenced using the BigDye Terminator v3.1 Cycle Sequencing Kit (ABI). Extension products were purified with Sephadex G-50 (Amersham), and resolved on an ABI 3730 DNA Analyzer. The software Sequencher 4.5 (Gene Codes Corp.) was used to edit chromatograms and assemble contigs. Gene identity was established by homology to GenBank entries with BLAST [46]. Direct sequences for elephants and consensus sequences for mammoths generated for *FGF5 *and *KRTHAP1 *have been deposited in GenBank [GenBank:FJ755444-FJ755451].

### Protein sequence analysis and structural prediction

Sequences were collected from the NCBI and the ENSEMBL databases using both keyword and homology searches. Multiple protein sequence alignments were performed using MAFFT 6 (E-INS-i algorithm; scoring matrix: BLOSUM 62; gap opening penalty: 1.53; gap extension penalty: 0.00) [47]. Pairwise alignments were performed using the Smith-Waterman algorithm [48]. N- and O-glycosylation sites were predicted using the NetNGlyc 1.0 and NetOGlyc 3.1 webservers [49]. The signal peptides were predicted using the SignalP 3.0 server [50]. The effects of mutations on protein function were predicted using the SIFT [51] and POLYPHEN programs [52]. Tests on protein stability and secondary structure predictions were performed using the MuPro  and SSPro8  webservers [53,54]. The PDB database  was searched to check whether the FGF5 structure has been experimentally resolved, but with negative results. For this reason, homology modeling and fold recognition were performed using the SWISS-MODEL [55] and PHYRE [56] web servers. Both programs identified the human FGF9 structure [PDB:1IHK] as the best candidate (most similar; E-value = 10^-45^) to build a structural model of FGF5. Therefore, the mammoth, elephant and human FGF5 proteins were modeled by using the human FGF9 as template. Pairwise structural alignments and model structural superimposition was performed using the SSAP [57,58] and DaliLite [59] webservers. Tertiary structure figures were generated using PyMol (DeLano Scientific; ).

FGF5 sequences from therian (placental and marsupial) mammals were aligned using MAFFT 6 (G-INS-i algorithm with JTT200 scoring matrix; gap opening penalty: 1.53; gap extension penalty: 0.00), and examined for residue variation using the FINGERPRINT web server [60]. The phylogenetic relationships among FGF5 sequences were examined in a maximum likelihood framework in RAxML 7.0.4 [61] using the best-fit JTT protein substitution matrix [62] with empirical residue frequencies and among-site rate heterogeneity modeled with Γ with four classes [63], after comparing the log-likelihood of all substitution models available in RAxML.

### Genome project sequences

Elephant sequences of *KRTHAP1*, *KRT25*, *KRT27 *AND *KRT83 *were identified in the NCBI *Loxodonta africana *genome Trace Archives  using MegaBlast [64]. Human or chimpanzee *KRTHAP1 *exon sequences were used as queries and obtained from GenBank [GenBank:AJ401054 and Y16795] or from the UCSC Genome Browser [65] (Human March 2006 [hg18] assembly). Elephant trace files obtained by matches to primates were themselves used as queries against the elephant genomic trace files, to obtain additional elephant sequences, with the process repeated to obtain further upstream and downstream elephant traces and sequences. Mammoth sequences were obtained from the mammoth genome project BLAST server [15]. Mammoth sequences with a score above 100 were used. The mammoth and elephant sequences were also verified using a BLAT search [66] against human sequences on the UCSC website to verify the identity of the locus.

### Transcription factor binding site and rare codon analyses

Transcription factor binding sites of promoter regions were predicted using TFSEARCH  that uses the TRANSFAC database [18]. The tRNA effect of the guanine-to-adenine mammoth-specific nucleotide substitution was examined using RARE CODON CALTOR .

## Authors' contributions

SF, KS, SH, MT, and ADG performed ancient DNA extractions, PCR and sequencing experiments. ALR and YI performed all modern elephant DNA work. NN, SOK and YI performed the bioinformatic and phylogenetic analyses and contributed to the writing of the manuscript. NN performed the protein comparison and structural modeling analysis. GB provided mammoth samples and morphological information. ALR and ADG designed the study, contributed to the experimental work and analysis and wrote the manuscript (with contributions from the others).

## Supplementary Material

Additional file 1 **Supplementary information for laboratory and analytical procedures**. Ancient DNA laboratory procedures, elephant and mammoth *FGF5 *primer sequences, FGF5 sequence properties and phylogenetic analysis, and mammoth *FGF5 *clone sequences. Click here for file
