# Supplementary material for: Genetic variation at hair length candidate genes in elephants and the extinct woolly mammoth
Source: BMC Evol Biol. 2009 Sep 11;9:232. doi: 10.1186/1471-2148-9-232 (PMC2754481; doi:10.1186/1471-2148-9-232)
Supplement: Additional file 1 — Supplementary information for laboratory and analytical procedures. Ancient DNA laboratory procedures, elephant and mammoth FGF5 primer sequences, FGF5 sequence properties and phylogenetic analysis, and mammoth FGF5 clone sequences. [file 1471-2148-9-232-S1.pdf]

ADDITIONAL FILE 1

Supplementary information for laboratory and analytical procedures.

Genetic variation at hair length candidate genes in elephants and the extinct woolly mammoth

Alfred L. Roca[1]*, Yasuko Ishida[1], Nikolas Nikolaidis[2], Sergios-Orestis Kolokotronis[3], Stephen Fratpietro[4], Kristin Stewardson[4], Shannon Hensley[5], Michele Tisdale[5], Gennady Boeskorov[6] and Alex D. Greenwood[5]*

[1] Department of Animal Sciences, University of Illinois at Urbana-Champaign, Urbana, IL 61801, USA
[2] Department of Biological Science, College of Natural Sciences and Mathematics, California State University at Fullerton, Fullerton, CA 92834, USA
[3] Sackler Institute for Comparative Genomics, American Museum of Natural History, New York, NY 10024, USA
[4] Paleo-DNA Laboratory, Lakehead University, Thunder Bay, ON P7B 5E1, Canada
[5] Department of Biological Sciences, Old Dominion University, Norfolk, VA 23529, USA
[6] Institute for Diamond and Precious Metals Geology, Siberian Branch of Russian Academy of Sciences, Yakutsk, Russian Federation

*Corresponding authors

E-mail addresses: A.L. Roca, roca@illinois.edu; Y. Ishida, yishida@illinois.edu; N. Nikolaidis, nnikolaidis@fullerton.edu; S.O. Kolokotronis, koloko@amnh.org; S. Frapietro, swfratpi@lakeheadu.ca; K. Stewardson, kmstewar@lakeheadu.ca; S. Hensley, shens003@odu.edu; M. Tisdale, mtisdoo3@odu.edu; G. Boeskorov, gboeskorov@yandex.ru; A.D. Greenwood, agreenwo@odu.edu

ADDITIONAL FILE 1, TABLE OF CONTENTS

Additional Text: Ancient DNA methods, mammoth mtDNA and exon 2	Pages 3-7
Additional Table S1: Elephant *FGF5* primer sequences	Page 8
Additional Table S2: Elephant *FGF5* primer combinations tested	Page 9
Additional Figures S1-S4: Primers for woolly mammoth *FGF5*	Pages 10-14
Additional Figure S5: Graphical depiction of FGF5 amino acid variation	Pages 15-17
Additional Figure S6: FGF5 protein maximum likelihood phylogram	Pages 18-19
Additional Figures S7-S10: Clone sequences for mammoth *FGF5*	Pages 20-54

Additional Text

Details of protocols for ancient DNA

Extractions of mammoth samples in Norfolk were carried out in a room dedicated to ancient DNA work in a CleanSpot PCR hood (Coy Laboratory) following an established protocol [1]. Approximately 0.5 gram of drilled bone powder was used per extraction. The room had never been previously used for molecular biological work. Separating rooms used for processing ancient DNA samples and performing modern molecular biological investigations is a useful way of minimizing contamination risk [2]. Amplified PCR products never entered the clean room nor did modern DNA. Extraction of DNA from the bone samples was done using GeneClean Ancient DNA Kits (MP Biomedicals) according to manufacturer's instructions. Mock extractions were performed to control for contamination introduced during extraction.

PCR amplifications were performed at least twice per primer pair. PCR amplification was performed for 40 cycles using HiFi Supermix (Invitrogen) which is known to perform well on ancient DNA extracts [3]. Annealing temperatures were chosen based on the Tm of the primers. All PCR products were cloned into T overhang vectors, transformed into competent bacteria, with positives colonies identified by colony PCR using M13 forward and reverse primers and multiple clones per PCR product sequenced using T7 or SP6 primers (Functional Biosciences, Inc.). Direct sequencing can lead to an erroneous sequence due to contamination and DNA damage in the extract. Cloning and sub-sampling individual representative amplified sequences provides a better representation of the original template amplified [4], therefore none of the consensus sequences generated in this study were determined from direct sequencing.

All pre-amplification work in Thunder Bay was performed in a 'Clean Lab'. Only ancient and degraded samples with low-copy number are allowed in the Clean Lab area. This lab is physically separated from post-amplification analysis, free from PCR product, slight positive pressure, surface sterilized weekly with a thorough monthly sterilization, and monitored quarterly to ensure the absence of modern DNA contamination. The outer surface of the container holding the tooth and bone were surface sterilized with 10% bleach, irradiated with UV light for 20 minutes, then passed

into a clean laboratory area. Preventative measures were taken to reduce the introduction of modern DNA to this area by wearing protective gear: a Tyvek™ suit (Kepler), hair net, face mask, an extra pair of Tyvek™ sleeves (Kepler), inner pair of nitrile gloves, outer pair of latex gloves, and safety glasses. In addition, once suited up, each analyst entered an air shower designed to remove any residual surface particulates from the external surface of the Tyvek™ suit before entering the Clean Lab.

The bone and tooth sample were surface sterilized with successive washes of 10% bleach, sterilized water, and 70% ethanol (Fisher). A Dremel™ tool was used with separate bits to cut a small portion of sample. These samples were separately milled into a fine powder using a Retsch mixer mill. Approximately 200 mg of powder was placed into 2.0 mL tubes. The extraction buffer was prepared using a modified protocol [5]: 1.5 mL of 0.5M EDTA (Sigma), 75 µL of 20% N-lauroylsarcosine (Sigma), and 40 µL of Proteinase K (Qiagen). A blank tube was also prepared containing extraction buffer only to act as a negative control. The tubes were placed onto an Eppendorf Thermomixer-R to incubate overnight at 56°C and 1000 rpm.

Next, the sample tubes were centrifuged for 5 minutes at 13,000 rpm and the aqueous extract transferred to a sterile 15 mL tube; 400 µL of phenol (Sigma) and 400 µL of chloroform:isoamyl alcohol (24:1, v/v) (Sigma) were added directly to each 15 mL tube. The tubes were vortexed for 1 minute then centrifuged for 5 minutes at maximum speed. The extract in each tube separated into two layers. The top layer was transferred with a pipette into a sterile 15 mL tube and the above process repeated with this layer. The bottom layer was discarded. After transferring the top layer to another sterile 15 mL tube for the second time, 800 µL of chloroform:isoamyl alcohol (24:1, v/v) was added. The tubes were vortexed for 1 minute then centrifuged for 5 minutes at maximum speed. The top layer was transferred to another sterile 15 mL tube. To each tube was added a 10% volume of 3M sodium acetate (Sigma) and 2.5 times the volume of cold anhydrous ethanol (Commercial Alcohols). These tubes were placed into a -20°C freezer overnight to allow precipitation of product. The next day, the tubes were centrifuged for 5 minutes at maximum speed. The supernatant was discarded. A volume of 1.5 mL of 95% ethanol (Commercial Alcohols) was added to each tube, the tubes vortexed to resuspend the precipitate and centrifuged for 10 minutes at maximum speed. The supernatant was

discarded and the precipitate allowed to dry for 1 hour. After this time the precipitate was resuspended in 100 µL TE buffer (10 mM Tris-HCl, pH 8.0, 10 mM EDTA) and purified additionally with Micro Bio-Spin 30 Chromatography Columns (Biorad).

The samples were amplified using Platinum® *Taq* DNA Polymerase (Invitrogen), 25 µL reaction: 2.5 µL 10X PCR buffer (minus Mg), 0.5 µL dNTP (0.25mM each, Invitrogen), 1.0 µL 50 mM $MgCl_2$, 0.1µL Platinum® *Taq* DNA Polymerase (5U/µL), 0.5 µL primer mix (0.1 µM each), 0.5 µL 20 mg/mL BSA (Fermentas) on an Eppendorf Mastercycler™. A hot start of 94°C for 2 minutes was used followed by 50 cycles of 94°C for 30 seconds denaturation, 47-65°C for 1 minute annealing, and 72°C for 2 minutes extension. 5 µL of each PCR product was mixed with 3 µL of 6X dye and resolved using 6% polyacrylamide gel electrophoresis run at 118V for 45 minutes. Ultra low range (Fermentas) size ladder was run in tandem with the PCR products. The 6% PAGE gel was stained with 0.4 µg/mL ethidium bromide (Sigma) for visualization of successful DNA amplification. Successful PCR products were purified using the QIAquick PCR purification kit (Qiagen) as per manufacturer's protocol. Colony PCR and sequencing were performed as in Norfolk.

Indigirka mammoth mtDNA clade

Recent genetic analysis of woolly mammoth populations suggests that there are two distinct mtDNA clades of mammoths with "Clade II" having become extinct much earlier than "Clade I" or the species as a whole. Whether Clade I and Clade II mammoths differ in phenotype is not clear. A 100 bp fragment of the mitochondrial DNA 16S rDNA was sequenced, which indicated that the Indigirka mammoth sample belonged to Clade I, the more common mtDNA clade of woolly mammoth [6].

Jarkov mammoth, exon 2 results

While the complete *FGF5* coding sequence was obtained for an Indigirka mammoth, few *FGF5* fragments could be retrieved from the Jarkov mammoth. All *FGF5* fragments retrieved were identical in sequence between the Indigirka mammoth and the Jarkov mammoth, with the exception of exon 2 amplicons from the Jarkov mammoth, which may have been due to contamination. Exon 2 was amplified in two

fragments. The first fragment was identical between all elephants and mammoth samples sequenced and differed from human at two positions. A second exon 2 fragment in the Jarkov mammoth was identical to human but differed from the elephants and the Indigirka mammoth at two positions. It seems likely that the second amplified fragment from the Jarkov mammoth represents contamination rather than variation among mammoth sequences. The difficulty in distinguishing between the alternatives is that exon 2 only differs between elephantids and human at four positions. The controls were negative and the same sequence was obtained from the Jarkov mammoth in all clones of two independent PCR reactions. This does not exclude the possibility that human contamination of the sample itself yielded the result. However, the other amplifications were identical to the Indigirka mammoth suggesting that contamination with human DNA was not present (Additional Figure S2). In addition, primers used to amplify mammoth sequences were designed based on elephant sequences which are quite divergent at the DNA level from human and thus not expected to co-amplify. The Jarkov mammoth failed to yield products for the majority of the PCR reactions successful with the Indigirka mammoth. Thus, it remains unclear whether there is among mammoth diversity in the *FGF5* gene. Given the complete sequencing from the Indigirka mammoth, and the limited success for the majority of the PCRs from the Jarkov mammoth, further analysis was restricted to the Indigirka sequence.

Additional References

1. Calvignac S, Terme JM, Hensley SM, Jalinot P, Greenwood AD, Hanni C: **Ancient DNA identification of early 20th century simian T-cell leukemia virus type 1**. *Mol Biol Evol* 2008, **25**(6):1093-1098.
2. Willerslev E, Cooper A: **Ancient DNA**. *Proc Biol Sci* 2005, **272**(1558):3-16.
3. Rohland N, Hofreiter M: **Comparison and optimization of ancient DNA extraction**. *Biotechniques* 2007, **42**(3):343-352.
4. Cooper A, Poinar HN: **Ancient DNA: do it right or not at all**. *Science* 2000, **289**(5482):1139.
5. Loreille OM, Diegoli TM, Irwin JA, Coble MD, Parsons TJ: **High efficiency DNA extraction from bone by total demineralization**. *Forensic Sci Int Genet* 2007, **1**(2):191-195.
6. Gilbert MT, Tomsho LP, Rendulic S, Packard M, Drautz DI, Sher A, Tikhonov A, Dalen L, Kuznetsova T, Kosintsev P *et al*: **Whole-genome shotgun sequencing of mitochondria from ancient hair shafts**. *Science* 2007, **317**(5846):1927-1930.

Table S1. *FGF5* oligonucleotide primer sequences used with extant elephant samples

Primer name	Primer sequence
**exon 1**	
FGF5X1-L1F (67)	ACCCCAGCGGCTGGAAGA
FGF5X1-L2F (62)	TCTCCCGAGGCTATGTCCA
FGF5X1-10F (60)	ATGAGCTTGTCCTTCCTCCTC
FGF5X1-60F (66)	CTCAGCGCCTGGGCTCAC
FGF5X1-80F (69)	TCGCCCCCAAAGGGCAAC
FGF5X1-170F (62)	GCAGCACCACGTCTTCCTC
FGF5X1-240F (61)	GAGCAGAGCAGCTTCCAGTG
FGF5X1-290F (61)	CCGGCAGCCTCTACTGC
FGF5X1-300F (62)	GTGGGCATCGGTTTCCAT
FGF5X1-350R (54)	CTTAACATACTGGCTTCGTG
FGF5X1-320R (61)	ACTTTGCCATCCGGGTAGAT
FGF5X1-290R (60)	AAACCGATGCCCACTCTG
FGF5X1-250R (65x)	CGAGGGGCTCCACTGGAA
FGF5X1-170R (63)	GGAGGGAGAGGAGGAAGACG
FGF5X1-100R (61)	GGTCCAGGTTGCCCTTTG
FGF5X1-50R (66)	CCCAGGCGCTGAGGATCA
FGF5X1-R1R (62)	GGTTTTGTAGAAGAGCGAGCAAC
FGF5X1-R2R (61)	CTCTCACCTAGCAACGGTTTTG
**exon 2**	
FGF5X2-L3F (58)	GTTTTATTTGGGATTTCTGTCATC
FGF5X2-L4F (62)	CAATAACAATGAGTCTGTGTTTTATTTGG
FGF5X2-360F (58)	GTATTTTGGAAATATTTGCTGTGTC
FGF5X2-370F (60)	GGAAATATTTGCTGTGTCTCAGG
FGF5X2-460R (59)	CACTTGCGTGGAGTTTTCC
FGF5X2-450R (59)	TGGAGTTTTCCTTTTTTTGACAT
FGF5X2-R3R (59)	CATCGTTAACAAATATAAAGTGGTTCTAC
FGF5X2-R4R (58x)	TTCTGTTTTAAAAACAGAAAATCTCC
**exon3**	
FGF5X3-L5F (59x)	CTATTTTCATCTCTTCTTTTTTTCTCC
FGF5X2-L6F (59x)	AGGTAGAAAATATTACCACTATTTTCATCTC
FGF5X3-480F (59)	TTACAGATGACTGCAAGTTCAGG
FGF5X3-520F (57)	AGAGATTTCAAGAAAACAGCTATAATAC
FGF5X3-540F (59)	CTCAGCAATACACAGAACTGAAAA
FGF5X3-640F (60)	AAACCCCAGCACATCTCTACC
FGF5X3-690F (62)	GAGCAGCCAGAACTTTCTTTCAC
FGF5X3-720F (60)	CCTGAAAAGAAAAAGCCACCTA
FGF5X3-760F (58)	GTTCCCCTTTCTGCACCT
FGF5X3-800R (62)	CCAAAGCGAAACTTGAGTCTGTATT
FGF5X3-750R (62)	AGGTGCAGAAAGGGGAACCT
FGF5X3-690R (58)	GAAAGAAAGTTCTGGCTGCTC
FGF5X3-610R (60)	CTGCAGCCTCGTTTAGCTTT
FGF5X3-560R (60x)	CGTTTTTTCAGTTCTGTGTATTGC
FGF5X3-520R (60)	GCGTAGGTATTATAGCTGTTTTCTTGA
FGF5X3-R6R (60)	TATACAACTCCTGAGGGGAAAGAA
FGF5X3-R5R (56)	CTCTGAAGACACCTATACAACTCC
**Promoter and 5'UTR**	
FGF5P-1F (60)	GGACAAGGAAAGAGGGGAAG
FGF5P-1R (60)	ATAGCCTCGGGAGAAAGAGG
FGF5P-2F (60)	TGGGAGAGGACAAGGAAAGA
FGF5P-2R (60)	GGAGAAAGAGGGGGAGAGAG
FGF5P-3R (61)	CGCCGCTTTGTGTACTCACT
FGF5-5'UTR-F (62)	CTTCGCAGGCGTGCACTA
FGF5-5'UTR-R (59)	CTGGGGTCTTAAGGGCATC

Primer names include direction (F-forward, R-reverse) and approximate location on exon; L and R are 5' and 3' to the exon
Parentheses indicate estimated Tm for primer, and lower case x indicates design issue as indicated by Primer3: http://frodo.wi.mit.edu/

Table S2. Primer combinations used for PCR and sequencing of extant elephant DNA

exon 1	FGF5X1-R1R (62)	FGF5X1-R2R (61)	FGF5X1-350R (54)	FGF5X1-320R (61)	FGF5X1-290R (60)	FGF5X1-250R (65x)	FGF5X1-170R (63)	FGF5X1-100R (61)	FGF5X1-50R (66)
FGF5X1-L1F (67)	Y	Y						Y	Y
FGF5X1-L2F (62)	Y	Y						Y	Y
FGF5X1-10F (60)							Y	Y	
FGF5X1-60F (66)						Y	Y		
FGF5X1-80F (69)						Y	Y		
FGF5X1-170F (62)					Y	Y			
FGF5X1-240F (61)	Y	Y	Y	Y					
FGF5X1-290F (61)	Y	Y							
FGF5X1-300F (62)	Y	Y							

exon 2	FGF5X2-R3R (59)	FGF5X2-R4R (58x)	FGF5X2-460R (59)	FGF5X2-450R (59)
FGF5X2-L3F (58)	Y	Y	Y	Y
FGF5X2-L4F (62)	Y	Y	Y	Y
FGF5X2-360F (58)	Y	Y	Y	Y
FGF5X2-370F (60)	Y	Y	Y	Y

exon3	FGF5X3-R5R (56)	FGF5X3-R6R (60)	FGF5X3-800R (62)	FGF5X3-750R (62)	FGF5X3-690R (58)	FGF5X3-610R (60)	FGF5X3-560R (60x)	FGF5X3-520R (60)
FGF5X3-L5F (59x)	Y	Y					Y	Y
FGF5X3-L6F (59x)	Y	Y					Y	Y
FGF5X3-480F (59)						Y	Y	
FGF5X3-520F (57)					Y	Y		
FGF5X3-540F (59)				Y	Y	Y		
FGF5X3-640F (60)			Y	Y				
FGF5X3-690F (62)		Y	Y					
FGF5X3-720F (60)	Y	Y						
FGF5X3-760F (58)	Y	Y						

promoter and 5'UTR	FGF5P-1R (60)	FGF5P-2R (60)	FGF5-5'UTR-R (59)
FGF5P-1F (60)	Y	Y	
FGF5P-2F (60)	Y	Y	
FGF5-5'UTR-F (62)			Y

Additional figures S1-S4 – Elephant sequences are used to show the positions of the overlapping primer pairs used to generate the sequences from the woolly mammoth for *FGF5*.

The PCR primers are shaded in grey and the product generated for each fragment is shown as a line above the sequence with the primer combination shown. The alignment uses the top sequence as a reference. Dots indicate identity to the reference and changes are shown as the base change. The primer sequences and combinations used are shown below the sequence alignments. Figures are in this order: exon 1, exon 2, exon 3 and promoter.

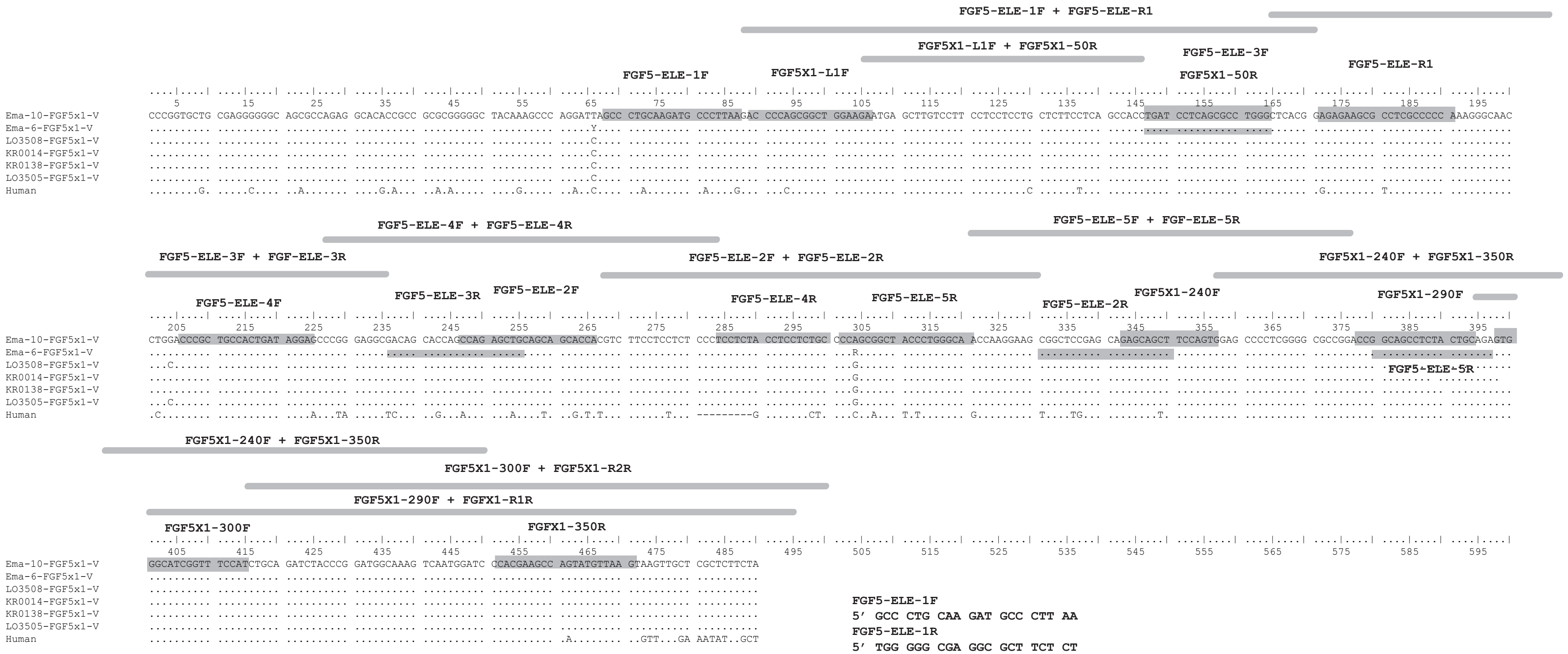

Primer combinations:
FGF5-ELE-1F + FGF5-ELE-R1
FGF5-ELE-3F + FGF-ELE-3R
FGF5-ELE-4F + FGF5-ELE-4R
FGF5-ELE-2F + FGF5-ELE-2R
FGF5-ELE-2F + FGF5-ELE-5R
FGF5-ELE-5F + FGF-ELE-5R
FGF5X1-240F + FGF5X1-350R
FGF5X1-300F + FGF5X1-R2R

FGF5-ELE-1F
5′ GCC CTG CAA GAT GCC CTT AA
FGF5-ELE-1R
5′ TGG GGG CGA GGC GCT TCT CT
FGF5-ELE-2F
5′ CCA GAG CTG CAG CAG CAC CAC
FGF5-ELE-2R
5′ AGC TGC TCT GCT CGG AGC CG
FGF5-ELE-3F
5′ TGA TCC TCA GCG CCT GG G 3′
FGF5-ELE-3R
5′ CAG CTC TGG CTG GTG CTG TC 3′
FGF5-ELE-4F
5′ CCC GCT GCC ACT GAT AGG AG 3′
FGF5-ELE-4R
5′GCA GAG GAG GTA GAG GA
FGF5-ELE-5F
5′ CCG GCG GCT ACC CTG GGC AA 3′
FGF5-ELE-5R
5′ GTG CAG TAG AGG CTG CC 3′
FGF5X1-240F
5′ GAG CAG AGC AGC TTC CAG TG
FGF5X1-350R
5′ CTT AAC ATA CTG GCT TCG TG
FGF5X1-300F
5′ GTG GGC ATC GGT TTC CAT
FGF5X1-R2R
5′CTC TCA CCT AGC AAC GGT TTT G

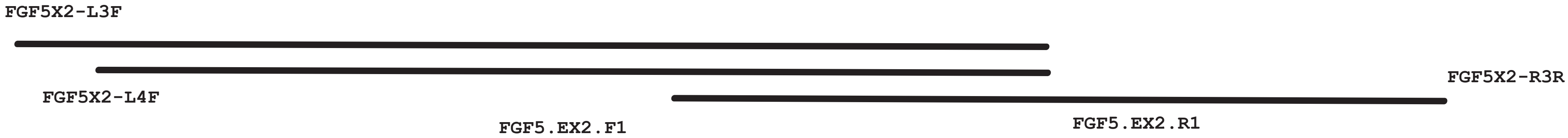


```
                  ....|....| ....|....| ....|....| ....|....| ....|....| ....|....| ....|....| ....|....| ....|....| ....|....| ....|....| ....|....| ....|
                      5         15         25         35         45         55         65         75         85         95         105        115        125
Ema-10-FGF5x2     GATTTCTGTC ATCCTAGGTA TTTTGGAAAT ATTTGCTGTG TCTCAGGGGA TCGTAGGAAT ACGAGGAGTT TTCAGCAACA AATTTTTAGC CATGTCAAAA AAAGGAAAAC TCCACGCAAG TGTAA
LO3505-FGF5x2     .......... .......... .......... .......... .......... .......... .......... .......... .......... .......... .......... .......... .....
KR0014-FGF5x2     .......... .......... .......... .......... .......... .......... .......... .......... .......... .......... .......... .......... .....
Human             .......... .........G .......... .......... .......... .T........ .......... .......... .......... G......... .......... ....T..... .....
```

FGF5X2-L3F
5′ GTT TAT TTG GGA TTT CTG TCA TC 3′
FGF5X2-L4F
5′ CAA TAA CAA TGA GTC TGT GTT TTA TTT GG 3′
FGF5.Ex2.F1
5′ TTG CTG TGT CTC AGG GGA TC 3′
FGF5X2-R3R
5′ CAT CGT TAA CAA ATA TAA AGT GGT TCT AC 3′
FGF5.EX2.R1
5′ GTT TTC CTT TTT TTG ACA TG 3′

Primer combinations:
FGF5X2-L3F or L4F + FGF5.EX2.R1
FGF5.Ex2.F1 + FGF5X2-R3R

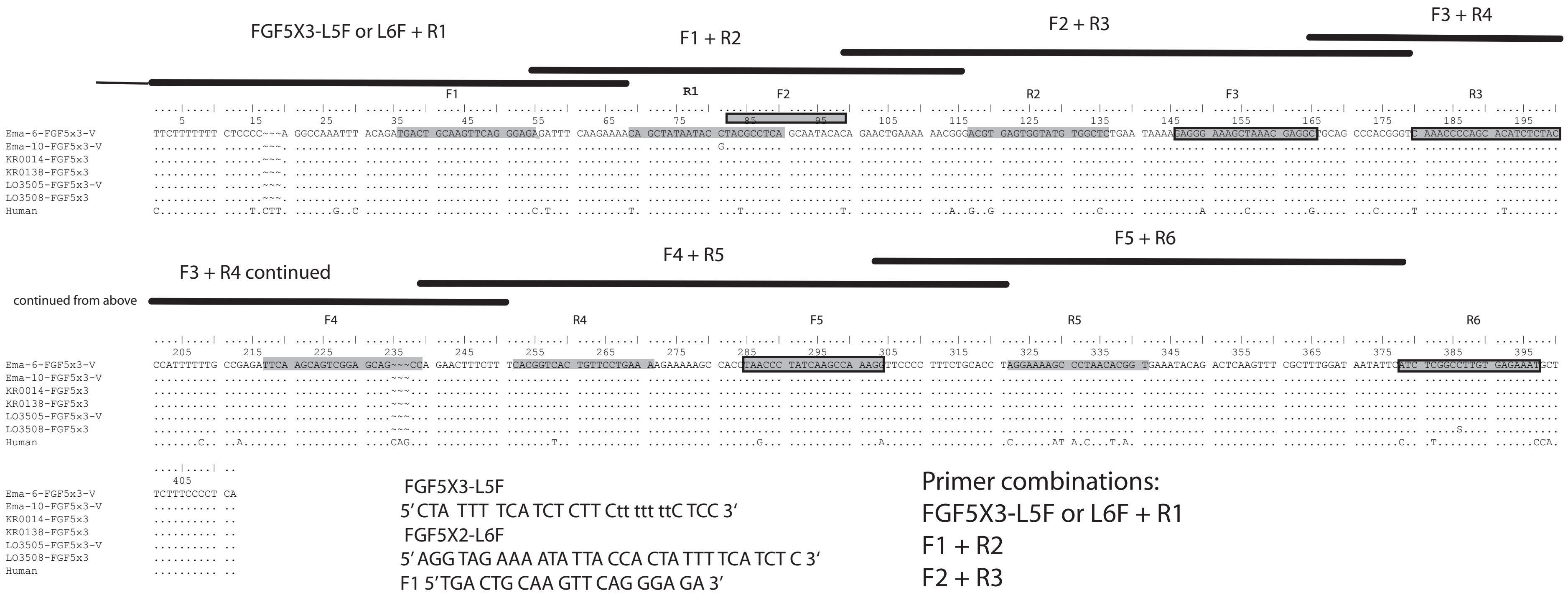

FGF5X3-L5F
5′ CTA TTT TCA TCT CTT Ctt ttt ttC TCC 3′
FGF5X2-L6F
5′ AGG TAG AAA ATA TTA CCA CTA TTT TCA TCT C 3′
F1 5′ TGA CTG CAA GTT CAG GGA GA 3′
F2 5′ TAC GCC TCA GCA ATA CAC 3′
F3 5′ GAG GGA AAG CTA AAC GAG GC 3′
F4 5′ TTC AAG CAG TCG GAG CAG CC 3′
F5 5′ TAA CCC TAT CAA GCC AAA GG 3′
R1 5′ AGG CGT AGG TAT TAT AGC TG 3′
R2 5′ GAG CCA CAT ACC ACT CAC GT 3′
R3 5′ TAG AGA TGT GCT GGG GT T TG 3′
R4 5′ TTT CAG GAA CAG TGA CCG TG 3′
R5 5′ ACC GTG TTA GGG CTT TTC CT 3′
R6 5′ ATT TCT CAC AAG GCC GAG AT 3′

Primer combinations:
FGF5X3-L5F or L6F + R1
F1 + R2
F2 + R3
F3 + R4
F4 + R5
F5 + R6

FGF5-PROM-L1 + FGF5-PROM-R1

FGF5-PROM-L2 + FGF5-PROM-R2

FGF5-PROM-L3 + FGF5-PROM-R3

```
            5         15         25         35         45         55         65         75         85         95        105        115        125        135        145        155        165        175        185        195
Ema10   CAAGTTGCAA CTCTCCACAC CGGCCGCTAC CCGTGGGGGA CTCGATCGCT TCCGCTAGGC TGAATTGCGC GATGCGCTGG TCCTGGTTGG GGGTGGCCAG GCAGCGCGTG AGAACTGCTG GGGTGCGTGG CCCCGGTGTG CGCGCACCCA GAGTCTGGGT GCAGCTTCGC TCCGCCAGGT TCTCCTCCCA CTCACTCTCT
Ema6    .......... .......... .......... .......... .......... .......... .......... .......... .......... .......... .......... .......... .......... .......... .......... .......... .......... .......... .......... ..........
KR138   .......... .......... .C........ .......... .......... .......... .......... .......... .......... .......... .......... .......... .......... .......... G......... .......... .......... ........C. .......... ..........
KR14    .......... .......... .C........ .......... .......... .......... .......... .......... .......... .......... .......... .......... .......... .......... G......... .......... .......... ........C. .......... ..........
LO3508  T......... .......... .C........ .......... .......... .......... .......... .......... .......... .......... .......... .......... .......... .......... G......... .......... .......... ........C. .......... ..........
```

FGF5-PROM-L3 + FGF5-PROM-R3 continued

FGF5-PROM-L4 + FGF5-PROM-R4

FGF5-PROM-L5 + FGF5-PROM-R5

```
          205        215        225        235        245        255        265        275        285        295        305        315        325        335        345        355        365        375        385        395
Ema10   CCCATCTCCT CCCTCCTCTA AACACCCCGC CCCTGGGGCT GCGGGGCTCC CTCTATTTAC CGGCCAGTGA GTACACAAAG CGGCGGGTGA GGGGAAGCTT CGCAGGCGTG CACTAAGCAG TGAGATCACT GGCGTTATAA ATATCCTGGT GCCAGAGCCG AGATCCGCTC GGGTGGCCTC TCTCTCCCCC TCTTTCTCCC
Ema6    .......... .......... .......... .......... .......... .......... .......... .......... .......... .......... .......... .......... .......... .......... .......... .......... .......... .......... .......... ..........
KR138   .......... .......... .......... .......... .......... .......... .......... .......... .......... .......... .......... .......... .......... .......... .......... .......T.. .......... .......... .......... ..........
KR14    .......... .......... .......... .......... .......... .......... .......... .......... .......... .......... .......... .......... .......... .......... .......... .......T.. .......... .......... .......... ..........
LO3508  .......... .......... .......... .......... .......... .......... .......... .......... .......... .......... .......... .......... .......... .......... .......... .......... .........G .......... .......... ..........
```

FGF5-PROM-L5 + FGF5-PROM-R5 continued

```
          405        415        425        435        445        455        465        475        485
Ema10   GAGGCTATGT CCACCCGGTG CTGCGAGGGG GGCAGCGCCA GAGGCACACC GCCGCGCGGG GGCTACAAAG CCCAGGATTA GCCCTGCAA
Ema6    .......... .......... .......... .......... .......... .......... .......... ........Y. .........
KR138   .......... .......... .......... .......... .......... .......... .......... ........C. .........
KR14    .......... .......... .......... .......... .......... .......... .......... ........C. .........
LO3508  .......... .......... .......... .......... .......... .......... .......... ........C. .........
```

FGF5-PROM-L1
5' TGT TGT CGA GCA CCC ATT TC
FGF5-PROM-L2
5' AAT TGC GCG ATG CGC TGG TC
FGF5-PROM-L3
5' GGT GCA GCT TCG CTC CGC CA
FGF5-PROM-L4
5' GGC TCC CTC TAT TTA CCG
FGF5-PROM-L5
5' GCA GTG AGA TCA CTG GCG
FGF5-PROM-R1
5' TCT CAC GCG CTG CCT GGC CA
FGF5-PROM-R2
5' AGA GAG TGA GTG GGA GGA GA
FGF5-PROM-R3
5' GCC GCT TTG TGT ACT CAC TG
FGF5-PROM-R4
5' CTC TGG CAC CAG GAT ATT
FGF5-PROM-R5
5' GGT GTG CCT CTG GCG CTG

Primer combinations:
FGF5-PROM-L1 + FGF5-PROM-R1
FGF5-PROM-L2 + FGF5-PROM-R2
FGF5-PROM-L3 + FGF5-PROM-R3
FGF5-PROM-L4 + FGF5-PROM-R4
FGF5-PROM-L5 + FGF5-PROM-R5

Additional figure S5 – Graphical depiction of FGF5 amino acid variation along the MAFFT alignment carried out with the G-INSi alignment algorithm with the JTT200 scoring matrix.

Composition shows the most frequent residue in each position. Heterogeneity shows the relative residue frequency at each site. Identity allows for a quick visual identification of variable sites. Heterozygosity represents the expected heterozygosity at each residue, i.e. the sum of the squared frequencies of each amino acid at a site. “Charges” indicates whether the most frequent residue at each position is acidic, basic, or uncharged. Hydrophobicity depicts the most frequent hydrophobic, hydrophilic, and charged residues at each position.

FGF5_Theria_only.GINSi.fasta 30 0.02454

Amino Acids

M S L F Gap H I A W G E K R
P Q T D N C Y V

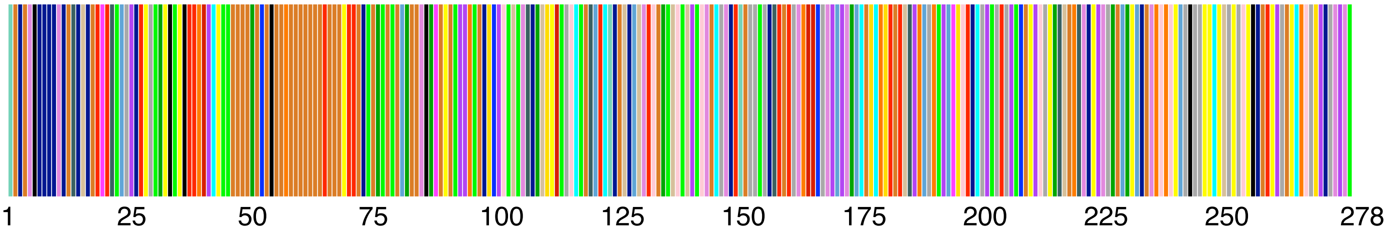

Heterogeneity

M S L F Gap I C T G H N A V
W P R Q Y E K D

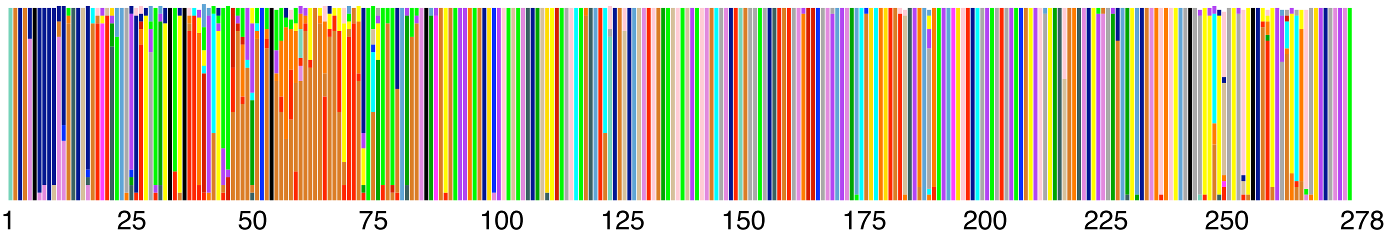

Identity

Constant Varied Gap

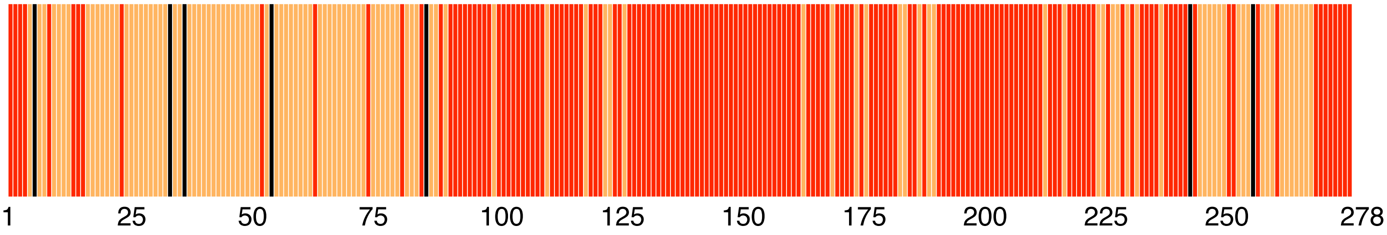

Heterozygosity

Gap 1 (Zero Heterozygosity) 0 (High Heterozygosity)

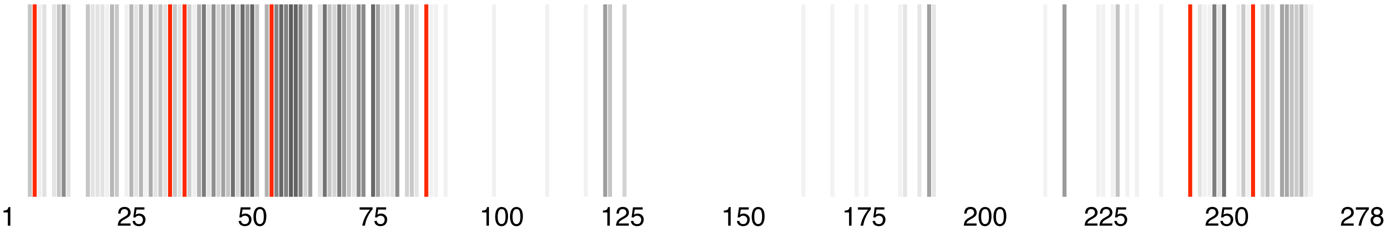

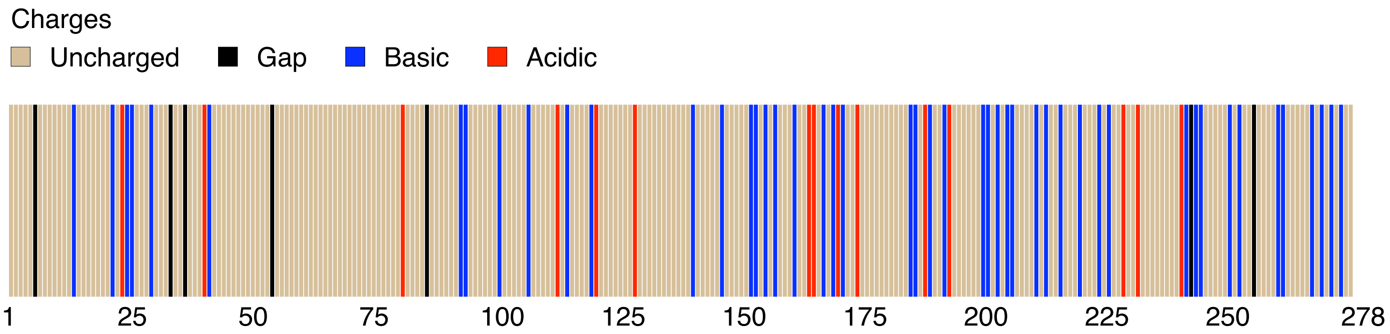
Charges
Uncharged
Gap
Basic
Acidic
1
25
50
75
100
125
150
175
200
225
250
278

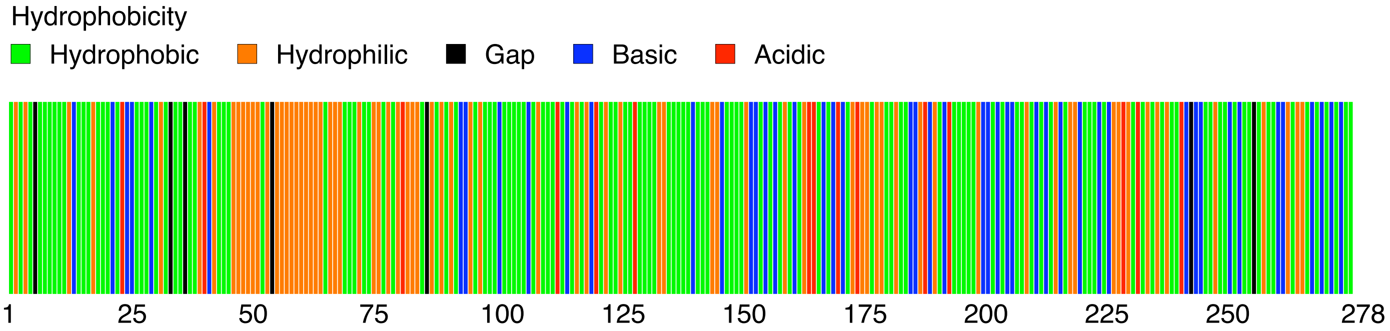
Hydrophobicity
Hydrophobic
Hydrophilic
Gap
Basic
Acidic
1
25
50
75
100
125
150
175
200
225
250
278

Additional figure S6 – FGF5 amino acid sequence maximum likelihood phylogram based on the G-INSi alignment algorithm with the JTT200 scoring matrix.

The opossum FGF5 amino acid sequence was used as outgroup. The scale bar denotes 0.05 substitutions/residue. α= 0.32417. Log-likelihood= -2594.951392.

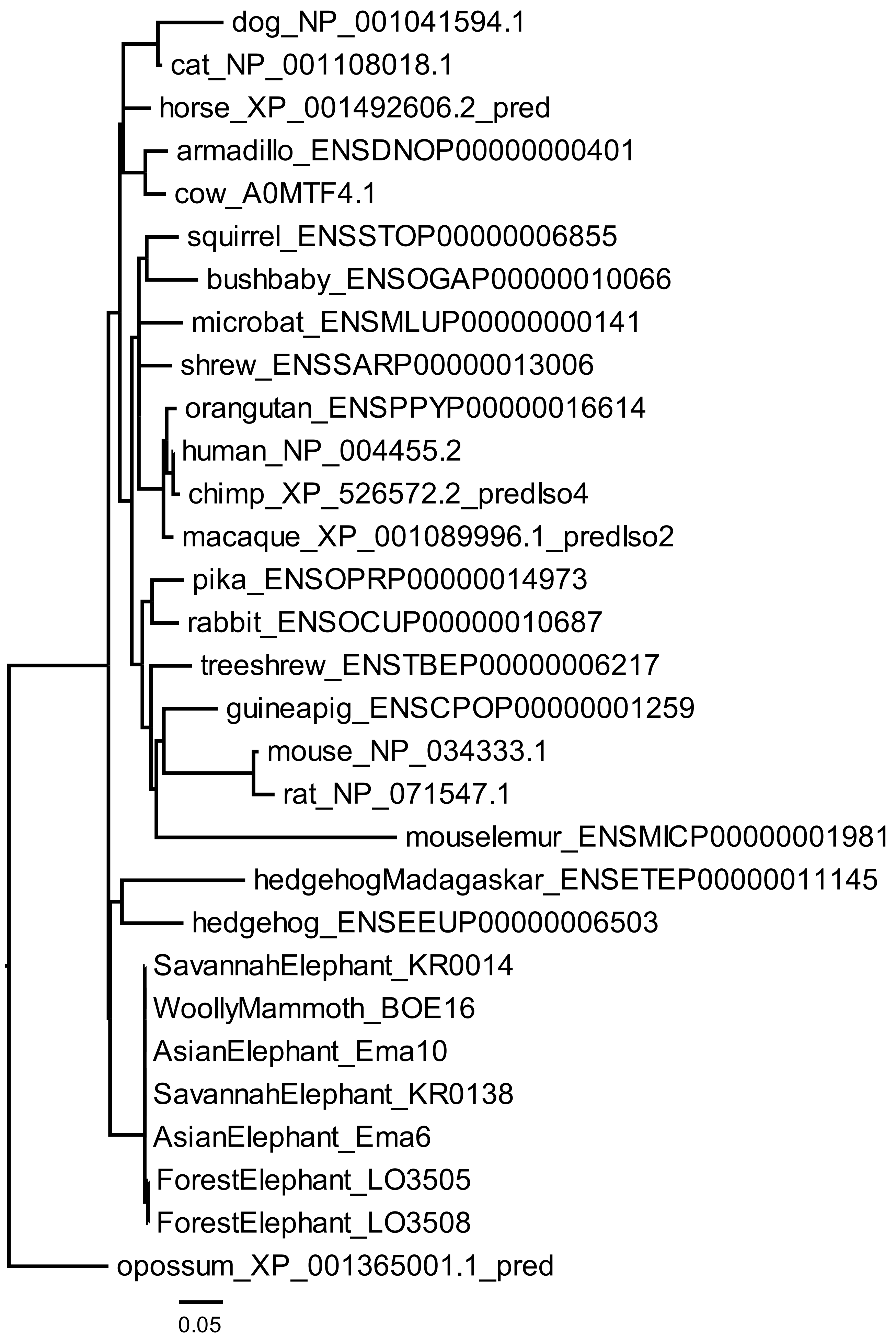

dog_NP_001041594.1
cat_NP_001108018.1
horse_XP_001492606.2_pred
armadillo_ENSDNOP00000000401
cow_A0MTF4.1
squirrel_ENSSTOP00000006855
bushbaby_ENSOGAP00000010066
microbat_ENSMLUP00000000141
shrew_ENSSARP00000013006
orangutan_ENSPPYP00000016614
human_NP_004455.2
chimp_XP_526572.2_predIso4
macaque_XP_001089996.1_predIso2
pika_ENSOPRP00000014973
rabbit_ENSOCUP00000010687
treeshrew_ENSTBEP00000006217
guineapig_ENSCPOP00000001259
mouse_NP_034333.1
rat_NP_071547.1
mouselemur_ENSMICP00000001981
hedgehogMadagaskar_ENSETEP00000011145
hedgehog_ENSEEUP00000006503
SavannahElephant_KR0014
WoollyMammoth_BOE16
AsianElephant_Ema10
SavannahElephant_KR0138
AsianElephant_Ema6
ForestElephant_LO3505
ForestElephant_LO3508
opossum_XP_001365001.1_pred
0.05

Additional figures S7-S10 – Clone sequences from which the mammoth *FGF5* sequences were derived are shown by these figures (each shown in one figure) in the following order: exon 1, 2 and 3 (x1, x2, x3) followed by the promoter.

Sequences are provided as an alignment in FASTA format. Modern elephant sequences determined by this study are also shown. “Ind” stands for the Indigirka mammoth. PCRs of the same fragment cloned and sequenced multiple times are designated A, B, C etc. The clone number is shown next to the letter e.g. A.1, A.2.

>Ema-6-FGF5x1-V
CCCGGTGCTGCGAGGGGGGCAGCGCCAGAGGCACACCGCCGCGCGGGGGCTACAAAGCCC
AGGATYAGCCCTGCAAGATGCCCTTAAGACCCCAGCGGCTGGAAGAATGAGCTTGTCCTT
CCTCCTCCTGCTCTTCCTCAGCCACCTGATCCTCAGCGCCTGGGCTCACGGAGAGAAGCG
CCTCGCCCCCAAAGGGCAACCTGGACCCGCTGCCACTGATAGGAGCCCGGGAGGCGACAG
CACCAGCCAGAGCTGCAGCAGCACCACGTCTTCCTCCTCTCCCTCCTCTACCTCCTCTGC
CCCRGCGGCTACCCTGGGCAACCAAGGAAGCGGCTCCGAGCAGAGCAGCTTCCAGTGGAG
CCCCTCGGGGCGCCGGACCGGCAGCCTCTACTGCAGAGTGGGCATCGGTTTCCATCTGCA
GATCTACCCGGATGGCAAAGTCAATGGATCCC~~ACGAAGCCAGTATG~~TTAAGTAAGT
TGCTCGCTCTTCTA--

>Ema-10-FGF5x1-V
CCCGGTGCTGCGAGGGGGGCAGCGCCAGAGGCACACCGCCGCGCGGGGGCTACAAAGCCC
AGGATTAGCCCTGCAAGATGCCCTTAAGACCCCAGCGGCTGGAAGAATGAGCTTGTCCTT
CCTCCTCCTGCTCTTCCTCAGCCACCTGATCCTCAGCGCCTGGGCTCACGGAGAGAAGCG
CCTCGCCCCCAAAGGGCAACCTGGACCCGCTGCCACTGATAGGAGCCCGGGAGGCGACAG
CACCAGCCAGAGCTGCAGCAGCACCACGTCTTCCTCCTCTCCCTCCTCTACCTCCTCTGC
CCCAGCGGCTACCCTGGGCAACCAAGGAAGCGGCTCCGAGCAGAGCAGCTTCCAGTGGAG
CCCCTCGGGGCGCCGGACCGGCAGCCTCTACTGCAGAGTGGGCATCGGTTTCCATCTGCA
GATCTACCCGGATGGCAAAGTCAATGGATCCC~~ACGAAGCCAGTATG~~TTAAGTAAGT
TGCTCGCTCTTCTA--

>LO3508-FGF5x1-V
CCCGGTGCTGCGAGGGGGGCAGCGCCAGAGGCACACCGCCGCGCGGGGGCTACAAAGCCC
AGGATCAGCCCTGCAAGATGCCCTTAAGACCCCAGCGGCTGGAAGAATGAGCTTGTCCTT
CCTCCTCCTGCTCTTCCTCAGCCACCTGATCCTCAGCGCCTGGGCTCACGGAGAGAAGCG
CCTCGCCCCCAAAGGGCAACCTGCACCCGCTGCCACTGATAGGAGCCCGGGAGGCGACAG
CACCAGCCAGAGCTGCAGCAGCACCACGTCTTCCTCCTCTCCCTCCTCTACCTCCTCTGC
CCCGGCGGCTACCCTGGGCAACCAAGGAAGCGGCTCCGAGCAGAGCAGCTTCCAGTGGAG
CCCCTCGGGGCGCCGGACCGGCAGCCTCTACTGCAGAGTGGGCATCGGTTTCCATCTGCA

GATCTACCCGGATGGCAAAGTCAATGGATCCC~~ACGAAGCCAGTATG~~TTAAGTAAGT
TGCTCGCTCTTCTA--

>KR0014-FGF5x1-V
CCCGGTGCTGCGAGGGGGGCAGCGCCAGAGGCACACCGCCGCGCGGGGGCTACAAAGCCC
AGGATCAGCCCTGCAAGATGCCCTTAAGACCCCAGCGGCTGGAAGAATGAGCTTGTCCTT
CCTCCTCCTGCTCTTCCTCAGCCACCTGATCCTCAGCGCCTGGGCTCACGGAGAGAAGCG
CCTCGCCCCCAAAGGGCAACCTGGACCCGCTGCCACTGATAGGAGCCCGGGAGGCGACAG
CACCAGCCAGAGCTGCAGCAGCACCACGTCTTCCTCCTCTCCCTCCTCTACCTCCTCTGC
CCCGGCGGCTACCCTGGGCAACCAAGGAAGCGGCTCCGAGCAGAGCAGCTTCCAGTGGAG
CCCCTCGGGGCGCCGGACCGGCAGCCTCTACTGCAGAGTGGGCATCGGTTTCCATCTGCA
GATCTACCCGGATGGCAAAGTCAATGGATCCC~~ACGAAGCCAGTATG~~TTAAGTAAGT
TGCTCGCTCTTCTA--

>KR0138-FGF5x1-V
CCCGGTGCTGCGAGGGGGGCAGCGCCAGAGGCACACCGCCGCGCGGGGGCTACAAAGCCC
AGGATCAGCCCTGCAAGATGCCCTTAAGACCCCAGCGGCTGGAAGAATGAGCTTGTCCTT
CCTCCTCCTGCTCTTCCTCAGCCACCTGATCCTCAGCGCCTGGGCTCACGGAGAGAAGCG
CCTCGCCCCCAAAGGGCAACCTGGACCCGCTGCCACTGATAGGAGCCCGGGAGGCGACAG
CACCAGCCAGAGCTGCAGCAGCACCACGTCTTCCTCCTCTCCCTCCTCTACCTCCTCTGC
CCCGGCGGCTACCCTGGGCAACCAAGGAAGCGGCTCCGAGCAGAGCAGCTTCCAGTGGAG
CCCCTCGGGGCGCCGGACCGGCAGCCTCTACTGCAGAGTGGGCATCGGTTTCCATCTGCA
GATCTACCCGGATGGCAAAGTCAATGGATCCCACGAAGCCAGTATGTTAAGTAAGTTGCT
CGCTCTTCTA--

>LO3505-FGF5x1-V
CCCGGTGCTGCGAGGGGGGCAGCGCCAGAGGCACACCGCCGCGCGGGGGCTACAAAGCCC
AGGATCAGCCCTGCAAGATGCCCTTAAGACCCCAGCGGCTGGAAGAATGAGCTTGTCCTT
CCTCCTCCTGCTCTTCCTCAGCCACCTGATCCTCAGCGCCTGGGCTCACGGAGAGAAGCG
CCTCGCCCCCAAAGGGCAACCTGCACCCGCTGCCACTGATAGGAGCCCGGGAGGCGACAG
CACCAGCCAGAGCTGCAGCAGCACCACGTCTTCCTCCTCTCCCTCCTCTACCTCCTCTGC
CCCGGCGGCTACCCTGGGCAACCAAGGAAGCGGCTCCGAGCAGAGCAGCTTCCAGTGGAG
CCCCTCGGGGCGCCGGACCGGCAGCCTCTACTGCAGAGTGGGCATCGGTTTCCATCTGCA
GATCTACCCGGATGGCAAAGTCAATGGATCCCACGAAGCCAGTATGTTAAGTAAGTTGCT
CGCTCTTCTA--

>Human
CCCGGTGCGGCGAGGCGGGCAGAGCCAGAGGCACGCAGCCGCACAGGGGCTACAGAGCCC
AGAATCAGCCCTACAAGATGCACTTAGGACCCCCGCGGCTGGAAGAATGAGCTTGTCCTT
CCTCCTCCTCCTCTTCTTCAGCCACCTGATCCTCAGCGCCTGGGCTCACGGGGAGAAGCG
TCTCGCCCCCAAAGGGCAACCCGGACCCGCTGCCACTGATAGGAACCCTAGAGGCTCCAG
CAGCAGACAGAGCAGCAGTAGCGCTATGTCTTCCTCTTCT---------GCCTCCTCCTC
CCCCGCAGCTTCTCTGGGCAGCCAAGGAAGTGGCTTGGAGCAGAGCAGTTTCCAGTGGAG
CCCCTCGGGGCGCCGGACCGGCAGCCTCTACTGCAGAGTGGGCATCGGTTTCCATCTGCA
GATCTACCCGGATGGCAAAGTCAATGGATCCCACGAAGCCAATATGTTAAGTGTTTTGGA
AATATTTGCT
>Ind.A1
~~~~~~
~~~~~~~~~~~~~~~~~~~~~~~~~~~GACCCCAGCGGCTGGAAGAATGAGCTTGTCCTT
CCTCCTCCTGCTCTTCCTCAGCCACCTGATCCTCAGCGCCTGGGCTCACGG
>Ind.A2
~~~~~~
~~~~~~~~~~~~~~~~~~~~~~~~~~~GACCCCAGCGGCTGGAAGAATGAGCTTGTCCTT
CCTCCTCCTGCTCTTCCTCAGCCACCTGATCCTCAGCGCCTGGGCTCACGG
>Ind.A3
~~~~~~
~~~~~~~~~~~~~~~~~~~~~~~~~~~GACCCCAGCGGCTGGAAGAATGAGCTTGTCCTT
CCTCCTCCTGCTCTTCCTCAGCCACCTGATCCTCAGCACCTGGGCTCACGG
>Ind.A4
~~~~~~
~~~~~~

~~~~~~~~~~~~~~~~~~~~~~~~~~~GACCCCAGCGGCTGGAAGAATGAGCTTGTCCTT
CCTCCTCCTGCTCTTCCTCAGCCACCTGATCCTCAGCACCTGGGCTCACGG
>Ind.A5
~~~~~~
~~~~~~~~~~~~~~~~~~~~~~~~~~~GACCCCAGCGGCTGGAAGAATGAGCTTGTCCTT
CCTCCTCCTGCTCTTCCTCAGCCACCTGATCCTCAGCGCCTGGGCTCACGG
>Ind.B1
~~~~~~
~~~~~~~~~~~~~~~~~~~~~~~~~~~GACCCCAGCGGCTGGAAGAATGAGCTTGTCCTT
CCTCCTCCTGCTCTTCCTCAGCCACCTGATCCTCAGCGCCTGGGCTCA~GG
>Ind.B2
~~~~~~
~~~~~~~~~~~~~~~~~~~~~~~~~~~GACCCCAGCGGCTGGAAGAATGAGCTTGTCCTT
CCTCCTCCTGCTCTTCCTCAGCCACCTGATCCTCAGCGCCTGGGCTCACGG
>Ind.B3
~~~~~~
~~~~~~~~~~~~~~~~~~~~~~~~~~~gaccccagcggctggaagaatgagcttgtcctt
cctcctcctgctcttcctcagccatctgatcctcagcgcctgggctcacgg
>Ind.B4
~~~~~~
~~~~~~~~~~~~~~~~~~~~~~~~~~~gaccccagcggctggaagaatgagcttgtcctt
cctcctcctgctcttcctcagccacctgatcctcagcgcctgggctcacgg
>Ind.C1
~~~~~~
~~~~~~~~~~~~~~~~~~~~~~~~~~~gaccccagcggctggaagaatgagcttgtcctt
cctcctcctgctcttcctcagccacctgatcctcagcgcctgggctcacgg
>Ind.C2
~~~~~~
~~~~~~~~~~~~~~~~~~~~~~~~~~~gaccccagcggctggaagaatgagcttgtcctt
cctcctcctgctcttcctcagccacctgatcctcagcgcctgggctcacgg
>Ind.C3
~~~~~~
~~~~~~~~~~~~~~~~~~~~~~~~~~~gaccccagcggctggaagaatgagcttgtcctt
cctcctcctgctcttcctcagccacctgatcctcagcgcctgggctcacgg
>Ind.C4
~~~~~~
~~~~~~~~~~~~~~~~~~~~~~~~~~~gaccccggcggctggaagaatgagtttgtcctt
ccttctcttgctcttcctcagccacctgatcctcagcgcctgggcactcgg
>Ind.C5
~~~~~~
~~~~~~~~~~~~~~~~~~~~~~~~~~~gaccccggcggctggaagaatgagcttgtcctt
cctccacctgctcttccacagccccctgatccacagcgcctgggcactcga
>Ind.A1
~~~~~~
~~~~~~
~~~~~~ctcacggagagaagcg

cctcgcccccaaagggcaacctggacccgctgccactgataggagcccgggaggc
>Ind.A2
~~~~~~
~~~~~~
~~~~~~ctcacagagagaagcg
cctcgcccccaaagggcaacctggacccgctgccactgataggagcccgggaggc
>Ind.A3
~~~~~~
~~~~~~
~~~~~~ctcacggagagaagcg
cctcgcccccaaagggcaacctggacccgctgccactgataggagcccgagaagc
>Ind.A4
~~~~~~
~~~~~~
~~~~~~ctcacggagagaagcg
cctcgcccccaaagggcaacctggacccgctgccactgataggagcccgggaggc
>Ind.A5
~~~~~~
~~~~~~
~~~~~~ctcacggagagaagcg
cctcgcccccaaagggcaacctggacccgctgccactgataggagcccgggaggc
>Ind.B1
~~~~~~
~~~~~~
~~~~~~ctcacggagagaagcg
cctcgcccccaaagggcaacctggacccgctgccactgataggagcccgggaggc
>Ind.B2
~~~~~~
~~~~~~
~~~~~~ctcacggagagaagcg
cctcacccccaaagggcaacctggacccgctgccactgataggagcccgggaggc
>Ind.A1
~~~~~~
~~~~~~
~~~~~~
~~~~~~cccgggaggcgacag
caccagccagagctgcagcagcaccacgtcttcctcctctccc
>Ind.A2
~~~~~~
~~~~~~
~~~~~~
~~~~~~cccgggaggcgacag
caccagccagagctgcagcagcaccacgtcttcctcctctccc
>Ind.A3
~~~~~~
~~~~~~

~~~~~~
~~~~~~cccgggaggcgacag
caccagccagagctgcagcaccaccacgtcttcctcctctccc
>Ind.A4
~~~~~~
~~~~~~
~~~~~~
~~~~~~cccgggaggcgacag
caccagccagagctgcagcaccaccacgtcttcctcctctccc
>Ind.A5
~~~~~~
~~~~~~
~~~~~~
~~~~~~cccgggaggcgacag
caccagccagagctgcagcagcaccacgtcttcctcctctccc
>Ind.A6
~~~~~~
~~~~~~
~~~~~~
~~~~~~cccgggaggcgacag
caccagccagagctgcagcagcaccacgtcttcctcctctccc
>Ind.A7
~~~~~~
~~~~~~
~~~~~~
~~~~~~cccgggaggcgacag
caccagccagagctgcagcagcaccacgtcttcctcctctccc
>Ind.A8
~~~~~~
~~~~~~
~~~~~~
~~~~~~cccgggaggcgacag
caccagccagagctgcagcagcaccacgtcttcctcctctccc
>Ind.A9
~~~~~~
~~~~~~
~~~~~~
~~~~~~cccgggaggcgacag
caccagccagagctgcagcagcaccacgtcttcctcctctccc
>Ind.B1
~~~~~~
~~~~~~
~~~~~~
~~~~~~cccgggaggcgacag
caccagccagagctgcagcagcaccacgtcttcctcctctccc
>Ind.B2

~~~~~~
~~~~~~
~~~~~~
~~~~~~cccgggaggcgacag
caccagccagagctgcagcagcaccacgtcttcctcctctccc
>Ind.B3
~~~~~~
~~~~~~
~~~~~~
~~~~~~cccgggaggcgacag
caccagccagagctgcagcagcaccacgtcttcctcctctccc
>Ind.C1
~~~~~~
~~~~~~
~~~~~~
~~~~~~CCTTTTTAGGCGACAG
CACCAGCCAGAGCTGCAGCAGCACCACGTCTTCCTCCTCTCCC
>Ind.C2
~~~~~~
~~~~~~
~~~~~~
~~~~~~CCCGGGAGGCGACAG
CACCAGCCAGAGCTGCAGCAGCACCACGTCTTCCTCCTCTCCC
>Ind.A1
~~~~~~
~~~~~~
~~~~~~
~~~~~~
~~~~~~~~~~~~~~~~~~~~~~~~~~~GTCTTCCTCCTCTCCCTCCTCTACCTCCTCTGC
CCCGGCGGCTACCCTGGTCAAACGAAGAAG
>Ind.A2
~~~~~~
~~~~~~
~~~~~~
~~~~~~
~~~~~~~~~~~~~~~~~~~~~~~~~~~GTCTTCCTCCTCTCCCTCCTCTACCTCCTCTGC
CCCGGCGGCTACCCTGGGCATCCAAGGAAG
>Ind.B1
~~~~~~
~~~~~~
~~~~~~
~~~~~~
~~~~~~~~~~~~~~~~~~~~~~~~~~~gtctttctcctctccctcctctacctcctctgc
cccggcggctaccctgggcaaccaaggaag
>Ind.C1
~~~~~~

~~~~~~
~~~~~~
~~~~~~
~~~~~~~~~~~~~~~~~~~~~~~~~~~gtcttcctcctctccctcctctatctcctctgc
cccggcggctaccctgggcaaccaaggaag
>Ind.C2
~~~~~~
~~~~~~
~~~~~~
~~~~~~
~~~~~~~~~~~~~~~~~~~~~~~~~~~gtcttcctcctctccctcctctacctcctctgc
cccggcggctaccctgggcaaccaaggaag
>Ind.C3
~~~~~~
~~~~~~
~~~~~~
~~~~~~
~~~~~~~~~~~~~~~~~~~~~~~~~~~gtcttcctcctctccctcctctacctcctctgc
cccggcggctaccctgggcaaccaaggaag
>Ind.C4
~~~~~~
~~~~~~
~~~~~~
~~~~~~
~~~~~~~~~~~~~~~~~~~~~~~~~~~gtcttcctcctctccctcctctacctcctctgc
cccggcggctaccctgggcaaccaaggaag
>Ind.A1
~~~~~~
~~~~~~
~~~~~~
~~~~~~
~~~~~~~~~~~~~~~~~~~~~~~~~~~GTCTTCCTCCTCTCCCTCCTCTACCTCCTCTGC
CCCGGCGGCTACCCTGGGCAACCAAGGAAGCGGCTCCGAGCAGAGCAGCTTCCAGTGGAG
CCCCTCGGGGCGCCGGACC
>Ind.A2
~~~~~~
~~~~~~
~~~~~~
~~~~~~
~~~~~~~~~~~~~~~~~~~~~~~~~~~GTCTTCCTCCTCTCCCTCCTCTACCTCCTCTGC
CCCGGCGGCTACCctgGGCAACCAAGGAAGCGGCTCCGAGCAGAGCAGCTTCCAGTGGAG
CCCCTCGGGGCGCCGGACC
>Ind.A3
~~~~~~
~~~~~~
~~~~~~

~~~~~~
~~~~~~~~~~~~~~~~~~~~~~~~~~~GTCTTCCTCCTCTCCCTCCTCTACCTCCTCTGC
CCCGGCGGCTACCCTGGGCAACCAAGGAAGCGGCTCCGAGCAGAGCAGCTTCCAGTGGAG
CCCCTCGGGGCGCCGGACC
>Ind.A1
~~~~~~
~~~~~~
~~~~~~
~~~~~~
~~~~~~
~~~~~~~~~~~~~~~~~~~~~ccaaggaagcgactccgagcagagcagcttccagtggag
cccctcggggcgccggacc
>Ind.A2
~~~~~~
~~~~~~
~~~~~~
~~~~~~
~~~~~~
~~~~~~~~~~~~~~~~~~~~~ccaaggaagcggctccgagcagagcagcttccagtgaag
cccctcggggcgccggacc
>Ind.A1
~~~~~~
~~~~~~
~~~~~~
~~~~~~
~~~~~~
~~~~~~gag
cccctcggggcgccggaccggcagcctctactgcagagtgggcatcggtttccatctgca
gatctacccggatggcaaagtcaatggatcc
>Ind.A2
~~~~~~
~~~~~~
~~~~~~
~~~~~~
~~~~~~
~~~~~~gag
cccctcggggcgccggaccggcagcctctactgcagagtgggcatcggtttccatctgca
gatctacccggatggcaaagtcaatggatcc
>Ind.A3
~~~~~~
~~~~~~
~~~~~~
~~~~~~
~~~~~~
~~~~~~gag
cccctcggggcgccggaccggcagcctctactgcagagtgggcatcggtttccatctgca

gatctacccggatggcaaagtcaatggatcc
>Ind.B1
~~~~~~
~~~~~~
~~~~~~
~~~~~~
~~~~~~
~~~~~~gag
cccctcggggcgccggaccggcagcctctactgcagagtgggcatcggtttccatctgca
gatctacccggatggcaaagtcaatggatcc
>Ind.B2
~~~~~~
~~~~~~
~~~~~~
~~~~~~
~~~~~~
~~~~~~gag
cccctcggggcgccggaccggcagcctctactgcagagtgggcatcggtttccatctgca
gatctacccggatggcaaagtcaatggatcc
>Ind.B3
~~~~~~
~~~~~~
~~~~~~
~~~~~~
~~~~~~
~~~~~~gag
cccctcggggcgccggaccggcagcctctactgcagagtgggcatcggtttccatctgca
gatctacccggatggcaaagtcaatggatcc
>Ind.B4
~~~~~~
~~~~~~
~~~~~~
~~~~~~
~~~~~~
~~~~~~gag
cccctcggggcgccggaccggcagcctctactgcagagtgggcatcggtttccatctgca
gatctacccggatggcaaagtcaatggatcc
>Ind.B5
~~~~~~
~~~~~~
~~~~~~
~~~~~~
~~~~~~
~~~~~~gag
cccctcggggcgccggaccggcagcctctactgcagagtgggcatcggtttccatctgca
gatctacccggatggcaaagtcaatggatcc

```
>Ind.A1
~~~~~~~~~~~~~~~~~~~~~~~~~~~~~~~~~~~~~~~~~~~~~~~~~~~~~~~~~~~~
~~~~~~~~~~~~~~~~~~~~~~~~~~~~~~~~~~~~~~~~~~~~~~~~~~~~~~~~~~~~
~~~~~~~~~~~~~~~~~~~~~~~~~~~~~~~~~~~~~~~~~~~~~~~~~~~~~~~~~~~~
~~~~~~~~~~~~~~~~~~~~~~~~~~~~~~~~~~~~~~~~~~~~~~~~~~~~~~~~~~~~
~~~~~~~~~~~~~~~~~~~~~~~~~~~~~~~~~~~~~~~~~~~~~~~~~~~~~~~~~~~~
~~~~~~~~~~~~~~~~~~~~~~~~~~~~~~~~~~~~~~~~~~~~~~~~~~~~~~~~~~~~
~~~~~~~~~~~~~~~~~~~~~~~~~~~~~~~~~~~~~~~~~~~~~~~~~~~~~~~ctgca
gatctacccggatggcaaagtcaatggatcccacgaagccagtatgttaagtaagttgct
cgctcttcta
>Ind.A2
~~~~~~~~~~~~~~~~~~~~~~~~~~~~~~~~~~~~~~~~~~~~~~~~~~~~~~~~~~~~
~~~~~~~~~~~~~~~~~~~~~~~~~~~~~~~~~~~~~~~~~~~~~~~~~~~~~~~~~~~~
~~~~~~~~~~~~~~~~~~~~~~~~~~~~~~~~~~~~~~~~~~~~~~~~~~~~~~~~~~~~
~~~~~~~~~~~~~~~~~~~~~~~~~~~~~~~~~~~~~~~~~~~~~~~~~~~~~~~~~~~~
~~~~~~~~~~~~~~~~~~~~~~~~~~~~~~~~~~~~~~~~~~~~~~~~~~~~~~~~~~~~
~~~~~~~~~~~~~~~~~~~~~~~~~~~~~~~~~~~~~~~~~~~~~~~~~~~~~~~~~~~~
~~~~~~~~~~~~~~~~~~~~~~~~~~~~~~~~~~~~~~~~~~~~~~~~~~~~~~~ctgca
gatctacccggatggcaaagtcaatggatcccacgaagccagtatgttaagtaagttgct
cgctcttcta
>Ind.A3
~~~~~~~~~~~~~~~~~~~~~~~~~~~~~~~~~~~~~~~~~~~~~~~~~~~~~~~~~~~~
~~~~~~~~~~~~~~~~~~~~~~~~~~~~~~~~~~~~~~~~~~~~~~~~~~~~~~~~~~~~
~~~~~~~~~~~~~~~~~~~~~~~~~~~~~~~~~~~~~~~~~~~~~~~~~~~~~~~~~~~~
~~~~~~~~~~~~~~~~~~~~~~~~~~~~~~~~~~~~~~~~~~~~~~~~~~~~~~~~~~~~
~~~~~~~~~~~~~~~~~~~~~~~~~~~~~~~~~~~~~~~~~~~~~~~~~~~~~~~~~~~~
~~~~~~~~~~~~~~~~~~~~~~~~~~~~~~~~~~~~~~~~~~~~~~~~~~~~~~~~~~~~
~~~~~~~~~~~~~~~~~~~~~~~~~~~~~~~~~~~~~~~~~~~~~~~~~~~~~~~ctgca
gatctacccggatggcgaagtcaatggatcccacgaagccagtatgttaagtaagttgct
cgctcttcta
>Ind.B1
~~~~~~~~~~~~~~~~~~~~~~~~~~~~~~~~~~~~~~~~~~~~~~~~~~~~~~~~~~~~
~~~~~~~~~~~~~~~~~~~~~~~~~~~~~~~~~~~~~~~~~~~~~~~~~~~~~~~~~~~~
~~~~~~~~~~~~~~~~~~~~~~~~~~~~~~~~~~~~~~~~~~~~~~~~~~~~~~~~~~~~
~~~~~~~~~~~~~~~~~~~~~~~~~~~~~~~~~~~~~~~~~~~~~~~~~~~~~~~~~~~~
~~~~~~~~~~~~~~~~~~~~~~~~~~~~~~~~~~~~~~~~~~~~~~~~~~~~~~~~~~~~
~~~~~~~~~~~~~~~~~~~~~~~~~~~~~~~~~~~~~~~~~~~~~~~~~~~~~~~~~~~~
~~~~~~~~~~~~~~~~~~~~~~~~~~~~~~~~~~~~~~~~~~~~~~~~~~~~~~~ctgca
gatctacccggatggcaaagtcaatggatcccacgaagccagtatgttaagtaagttgct
cgctctttta
>Ind.B2
~~~~~~~~~~~~~~~~~~~~~~~~~~~~~~~~~~~~~~~~~~~~~~~~~~~~~~~~~~~~
~~~~~~~~~~~~~~~~~~~~~~~~~~~~~~~~~~~~~~~~~~~~~~~~~~~~~~~~~~~~
~~~~~~~~~~~~~~~~~~~~~~~~~~~~~~~~~~~~~~~~~~~~~~~~~~~~~~~~~~~~
~~~~~~~~~~~~~~~~~~~~~~~~~~~~~~~~~~~~~~~~~~~~~~~~~~~~~~~~~~~~
~~~~~~~~~~~~~~~~~~~~~~~~~~~~~~~~~~~~~~~~~~~~~~~~~~~~~~~~~~~~
```

~~~~~~
~~~~~~ctgca
gatctacccggatggcaaagtcaatggatcccacgaagccagtatgttaagtaagttgct
cgctcttcta

>Ema-10-FGF5x2
GATTTCTGTCATCCTAGGTATTTTGGAAATATTTGCTGTGTCTCAGGGGATCGTAGGAAT
ACGAGGAGTTTTCAGCAACAAATTTTTAGCCATGTCAAAAAAAGGAAAACTCCACGCAAG
TGTAA
>LO3505-FGF5x2
GATTTCTGTCATCCTAGGTATTTTGGAAATATTTGCTGTGTCTCAGGGGATCGTAGGAAT
ACGAGGAGTTTTCAGCAACAAATTTTTAGCCATGTCAAAAAAAGGAAAACTCCACGCAAG
TGTAA
>KR0014-FGF5x2
GATTTCTGTCATCCTAGGTATTTTGGAAATATTTGCTGTGTCTCAGGGGATCGTAGGAAT
ACGAGGAGTTTTCAGCAACAAATTTTTAGCCATGTCAAAAAAAGGAAAACTCCACGCAAG
TGTAA
>Human
GATTTCTGTCATCCTAGGTGTTTTGGAAATATTTGCTGTGTCTCAGGGGATTGTAGGAAT
ACGAGGAGTTTTCAGCAACAAATTTTTAGCGATGTCAAAAAAAGGAAAACTCCATGCAAG
TGTAA
>Ind.A1
gatttctgtcatcctaggtattttggaaatatttgctgtgtctcaggggatcgtaggaat
acgaggagttttcagcaacaaatttttagc
>Ind.A2
gatttctgtcatcctaggtattttggaaatatttgctgtgtctcaggggatcgtaggaat
acgaggagttttcagcaacaaatttttagc
>Ind.A3
gatttctgtcatcctaggtattttggaaatatttgctgtgtctcaggggatcgtaggaat
acgaggagttttcagcaacaaatttttagc
>Ind.A1
~~~~~~~~~~~~~ctaggtattttggaaatatttgccgtgtctcaggggatcgtaggaat
acgaggagttttcagcaataaatttttagc
>Ind.A2
~~~~~~~~~~~~~ctaggtattttggaaatatttgctgtgtctcagggaatcgtaggaat
acgaggagttttcagcaacaaatttttagc
>Ind.A1
~~~~~~gtaggaat
acgaggagttttcagcaacaaatttttagccatgtcaaaaaaaggaaaactccacgcaag
tgtaa
>Ind.A2
~~~~~~gtaggaat
acgaggagttttcagcaacaaatttttagccatgtcaaaaaaaggaaaactccacgcaag
tataa
>Ind.A3
~~~~~~gtaggaat
acgaggagttttcagcaacaaatttttagccatgtcaaaaaaaggaaaactccacgcaag
tgtaa
>Ind.A4
~~~~~~gtaggaat
acgaggagttttcagcaacaaatttttagccatgtcaaaaaaaggaaaactccacgcaa

```
>Ind.A5
~~~~~~~~~~~~~~~~~~~~~~~~~~~~~~~~~~~~~~~~~~~~~~~~~~~~gtaggaat
acgaggagttttcagcaacacccttttagccatgtcaaaaaaaggaaaactccacgcaag
tgtaa
>Ind.A6
~~~~~~~~~~~~~~~~~~~~~~~~~~~~~~~~~~~~~~~~~~~~~~~~~~~~gtaggaat
acgaggagttttcagcaacaaatttttagccatgtcaaaaaaaggaaaactccacgcaag
tgtaa
>Ind.A7
~~~~~~~~~~~~~~~~~~~~~~~~~~~~~~~~~~~~~~~~~~~~~~~~~~~~gtaggaat
acgaggagttttcagcaacaaatttttagccatgtcaaaaaaaggaaaactccacgcaag
tgtaa
>Ind.B1
~~~~~~~~~~~~~~~~~~~~~~~~~~~~~~~~~~~~~~~~~~~~~~~~~~~~gtaagaat
acgaggagttttcagcaacaaatttttagccatgtcaaaaaaaagaaaactccacgcaag
tgtaa
>Ind.C1
~~~~~~~~~~~~~~~~~~~~~~~~~~~~~~~~~~~~~~~~~~~~~~~~~~~~gtaggaat
acgaggagttttcagcaacaaatttttagccatgtcaaaaaaaggaaaactccacgcaag
tgtaa
>Ind.C2
~~~~~~~~~~~~~~~~~~~~~~~~~~~~~~~~~~~~~~~~~~~~~~~~~~~~gtaggaat
acgaggagtcttcagcaacaaatttttagccatgtcaaaaaaaggaaaactccacgcaag
tgtaa
>Ind.C3
~~~~~~~~~~~~~~~~~~~~~~~~~~~~~~~~~~~~~~~~~~~~~~~~~~~~gtaggaat
acgaggagttttcagcaacaaatttttagccatgtcaaaaaaaggaaaactccacgcaag
tgtaa
>Ind.C4
~~~~~~~~~~~~~~~~~~~~~~~~~~~~~~~~~~~~~~~~~~~~~~~~~~~~gtaggaat
acgaggagttttcagcaacaaatttttagccatgtcaaaaaaaggaaaactccacgcaag
tgtaa
>Jarkov.A1
~~~~~~~~~~~~~ctaggtattttggaaatatttgctgtgtctcaggggatcgtaggaat
acgaggagttttcagcaacaaatttttagc
>Jarkov.A2
~~~~~~~~~~~~~ctaggtattttggaaatatttgctgtgtctcaggggatcgtaggaat
acgaggagttttcagcaacaaatttttagc
>Jarkov.A1
~~~~~~~~~~~~~~~~~~~~~~~~~~~~~~~~~~~~~~~~~~~~~~~~~~~~gtaggaat
acgaggagttttcagcaacaaatttttagcgatgtcaaaaaaaggaaaactccatgcaag
tgtaa
>Jarkov.A2
~~~~~~~~~~~~~~~~~~~~~~~~~~~~~~~~~~~~~~~~~~~~~~~~~~~~gtaggaat
acgaggagttttcagcaacaaatttttagcgatgtcaaaaaaaggaaaactccatgcaag
tgtaa
```

>Jarkov.A3
~~~~~~gtaggaat
acgaggagttttcagcaacaaatttttagcgatgtcaaaaaaaggaaaactccatgcaag
tgtaa
>Jarkov.A4
~~~~~~gtaggaat
acggggagttttcagcaacaaatttttagcgatgtcaaaaaaaggaaaactccatgcaag
tgtaa
>Jarkov.A5
~~~~~~gtaggaat
acgaggagttttcagcaacaaatttttagcgatgtcaaaaaaaggaaaactccatgcaag
tgtaa
>Jarkov.A6
~~~~~~gtaggaat
acgaggagttttcagcaacaaatttttagcgatgtcaaaaaaaggaaaactccatgcaag
tgtaa
>Jarkov.A7
~~~~~~gtaggaat
acgaggagttttcagcaacaaatttttagcgatgtcaaaaaaaggaaaactccatgcaag
tgtaa
>Jarkov.A8
~~~~~~gtaggaat
acgaggagttttcagcaacaaatttttagcgatgtcaaaaaaaggaaaactccatgcaag
tgtaa
>Jarkov.B1
~~~~~~gtaggaat
acgaggagttttcagcaacaaatttttagcgatgtcaaaaaaaggaaaactccatgcaag
tgtaa
>Jarkov.B2
~~~~~~gtaggaat
acgaggagttttcagcaacaaatttttagcgatgtcaaaaaaaggaaaactccatgcaag
tgtaa
>10B.6
gtaggaatacgaggagttttcagcaacaaatttttagccatgtcaaaaaaaggaaaactc
cacgcaagtgtaa

>Ema-6-FGF5x3-V
TTCTTTTTTTCTCCCC~~~AGGCCAAATTTACAGATGACTGCAAGTTCAGGGAGAGATTT
CAAGAAAACAGCTATAATACCTACGCCTCAGCAATACACAGAACTGAAAAAACGG~GACG
TGAGTGGTATGTGGCTCTGAATAAAAGAGGGAAAGCTAAACGAGGCTGCAGCCCACGGGT
CAAACCCCAGCACATCTCTACCCATTTTTTGCCGAGATTCAAGCAGTCGGAGCAG~~~CC
AGAACTTTCTTTCACGGTCACTG~TTCCTGAAAAGAAAAAGCCACCTAACCCTATCAAGC
CAAAGGTTCCCCTTTCTGCACCTAGGAAAAGCCCTAACACGGTGAAATACAGACTCAAGT
TTCGCTTTGGATAATATTCATCTCGGCCTTGTGAGAAATGCTTCTTTCCCCTCA
>Ema-10-FGF5x3-V
TTCTTTTTTTCTCCCC~~~AGGCCAAATTTACAGATGACTGCAAGTTCAGGGAGAGATTT
CAAGAAAACAGCTATAATACGTACGCCTCAGCAATACACAGAACTGAAAAAACGG~GACG
TGAGTGGTATGTGGCTCTGAATAAAAGAGGGAAAGCTAAACGAGGCTGCAGCCCACGGGT
CAAACCCCAGCACATCTCTACCCATTTTTTGCCGAGATTCAAGCAGTCGGAGCAG~~~CC
AGAACTTTCTTTCACGGTCACTG~TTCCTGAAAAGAAAAAGCCACCTAACCCTATCAAGC
CAAAGGTTCCCCTTTCTGCACCTAGGAAAAGCCCTAACACGGTGAAATACAGACTCAAGT
TTCGCTTTGGATAATATTCATCTCGGCCTTGTGAGAAATGCTTCTTTCCCCTCA
>KR0014-FGF5x3
TTCTTTTTTTCTCCCC~~~AGGCCAAATTTACAGATGACTGCAAGTTCAGGGAGAGATTT
CAAGAAAACAGCTATAATACCTACGCCTCAGCAATACACAGAACTGAAAAAACGG~GACG
TGAGTGGTATGTGGCTCTGAATAAAAGAGGGAAAGCTAAACGAGGCTGCAGCCCACGGGT
CAAACCCCAGCACATCTCTACCCATTTTTTGCCGAGATTCAAGCAGTCGGAGCAG~~~CC
AGAACTTTCTTTCACGGTCACTG~TTCCTGAAAAGAAAAAGCCACCTAACCCTATCAAGC
CAAAGGTTCCCCTTTCTGCACCTAGGAAAAGCCCTAACACGGTGAAATACAGACTCAAGT
TTCGCTTTGGATAATATTCATCTCGGCCTTGTGAGAAATGCTTCTTTCCCCTCA
>KR0138-FGF5x3
TTCTTTTTTTCTCCCC~~~AGGCCAAATTTACAGATGACTGCAAGTTCAGGGAGAGATTT
CAAGAAAACAGCTATAATACCTACGCCTCAGCAATACACAGAACTGAAAAAACGG~GACG
TGAGTGGTATGTGGCTCTGAATAAAAGAGGGAAAGCTAAACGAGGCTGCAGCCCACGGGT
CAAACCCCAGCACATCTCTACCCATTTTTTGCCGAGATTCAAGCAGTCGGAGCAG~~~CC
AGAACTTTCTTTCACGGTCACTG~TTCCTGAAAAGAAAAAGCCACCTAACCCTATCAAGC
CAAAGGTTCCCCTTTCTGCACCTAGGAAAAGCCCTAACACGGTGAAATACAGACTCAAGT
TTCGCTTTGGATAATATTCATCTCGGCCTTGTGAGAAATGCTTCTTTCCCCTCA
>LO3505-FGF5x3-V
TTCTTTTTTTCTCCCC~~~AGGCCAAATTTACAGATGACTGCAAGTTCAGGGAGAGATTT
CAAGAAAACAGCTATAATACCTACGCCTCAGCAATACACAGAACTGAAAAAACGG~GACG
TGAGTGGTATGTGGCTCTGAATAAAAGAGGGAAAGCTAAACGAGGCTGCAGCCCACGGGT
CAAACCCCAGCACATCTCTACCCATTTTTTGCCGAGATTCAAGCAGTCGGAGCAG~~~CC
AGAACTTTCTTTCACGGTCACTG~TTCCTGAAAAGAAAAAGCCACCTAACCCTATCAAGC
CAAAGGTTCCCCTTTCTGCACCTAGGAAAAGCCCTAACACGGTGAAATACAGACTCAAGT
TTCGCTTTGGATAATATTCATCTCGGCCTTGTGAGAAATGCTTCTTTCCCCTCA
>LO3508-FGF5x3
TTCTTTTTTTCTCCCC~~~AGGCCAAATTTACAGATGACTGCAAGTTCAGGGAGAGATTT
CAAGAAAACAGCTATAATACCTACGCCTCAGCAATACACAGAACTGAAAAAACGG~GACG
TGAGTGGTATGTGGCTCTGAATAAAAGAGGGAAAGCTAAACGAGGCTGCAGCCCACGGGT
CAAACCCCAGCACATCTCTACCCATTTTTTGCCGAGATTCAAGCAGTCGGAGCAG~~~CC
AGAACTTTCTTTCACGGTCACTG~TTCCTGAAAAGAAAAAGCCACCTAACCCTATCAAGC

CAAAGGTTCCCCTTTCTGCACCTAGGAAAAGCCCTAACACGGTGAAATACAGACTCAAGT
TTCGCTTTGGATAATATTCATCTCGGCSTTGTGAGAAATGCTTCTTTCCCCTCA
>Human
CTCTTTTTTTCTCCTCCTTAGGCCAAGTTCACAGATGACTGCAAGTTCAGGGAGCGTTTT
CAAGAAAATAGCTATAATACCTATGCCTCAGCAATACATAGAACTGAAAAAACAG~GGCG
GGAGTGGTATGTGGCCCTGAATAAAAGAGGAAAAGCCAAACGAGGGTGCAGCCCCCGGGT
TAAACCCCAGCATATCTCTACCCATTTTCTGCCAAGATTCAAGCAGTCGGAGCAGCAGCC
AGAACTTTCTTTCACGGTTACTG~TTCCTGAAAAGAAAAAGCCACCTAGCCCTATCAAGC
CAAAGATTCCCCTTTCTGCACCTCGGAAAAATACCAACTCAGTGAAATACAGACTCAAGT
TTCGCTTTGGATAATATTCCTCTTGGCCTTGTGAGAAACCATTCTTTCCCCTCA
>Ind.A1
ttctttttttctcccc~~~agaccaaatttacagatgactgcaagttcagggagagattt
caagaaaa
>Ind.A2
ttctttttttctcccc~~~aggccaaatttacagatgactgcaagttcagggagagattt
caagaaaa
>Ind.A3
ttctttttttctcccc~~~aggccaaatttacagatgactgcaagttcagggagagattt
caagaaaa
>Ind.A4
ttctttttttctcccc~~~aggccaaatttacagatgactgcaagttcagggagagattt
caagaaaa
>Ind.A5
ttctttttttctcccc~~~aggccaaatttacagatgactgcaagttcagggagagattt
caagaaaa
>Ind.A6
ttctttttttctcccc~~~aggccaaatttacagatgactgcaagttcagggagagattt
caagaaaa
>Ind.A7
ttcttttttt~ctcccc~~~aggccaaatttacagatgactgcaagttcagggagagattt
caagaaaa
>Ind.A8
ttctttttttctcccc~~~aggccaaatttacagatgactgcaagttcagggagagattt
caagaaaa
>Ind.B1
ttctttttttctcccc~~~aggccaaatttacagatgactgcaagttcagggagagattt
caagaaaa
>Ind.B2
ttctttttttctcccc~~~aggccaaatttacagatgactgcaagttcagggagagattt
caagaaaa
>Ind.B3
ttctttttttctcccc~~~aggccaaatttacagatgactgcaagttcagggagagattt
caagaaaa
>Ind.B4
ttctttttttctcccc~~~aggccaaatttacagatgactgcaagttcagggagagattt
caagaaaa

>Ind.B5
ttctttttttctcccc~~~aggccaaatttacagaagactgcaagttcagggagagattt
caagaaaa
>Ind.B6
ttctttttttctcccc~~~aggccaaatttacagatgactgcaagttcagggagagattt
caagaaaa
>Ind.B7
ttctttttttctcccc~~~aggccaaatttacagatgactgcaagttcagggagagattt
caagaaaa
>Ind.B8
ttctttttttctcccc~~~aggccaaatttacagatgactgcaagttcagggagagattt
caagaaaa
>Ind.A1
ttctttttttctcccc~~~aggccaaatttacagatgactgcaagttcagggagagattt
caagaaaa
>Ind.A2
ttctttttttctcccc~~~aggccaaatttacagatgactgctagttcagggagagattt
caagaaaa
>Ind.A3
ttctttttttctcccc~~~aggccaaatttacagatgactgcaagttcagggagagattt
caagaaaa
>Ind.A4
ttctttttttctcccc~~~aggccaaatttacagatgactgcaagttcagggagagattt
caagaaaa
>Ind.A5
ttctttttttctcccc~~~aggccaaatttacagatgactgcaagttcagggagagattt
caagaaaa
>Ind.A6
ttctttttttctcccc~~~aggccaaatttacagatgactgcaagttcagggagagattt
caagaaaa
>Ind.A7
ttctttttttctcccc~~~aggccaaatttacagatgactgcaagttcagggagagattt
caagaaaa
>Ind.A8
ttctttttttctcccc~~~aggccaaatttacagatgactgcaagttcagggagagattt
caagaaaa
>Ind.A1
~~~~~~gattt
caagaaaacagctataatacctacgcctcagcaatacacagaactgaaaaaacag~g
>Ind.A2
~~~~~~gattt
caagaaaacagctataatacctacgcctcagcaatacacagaactgaaaaaacag~g
>Ind.A3
~~~~~~gattt
caagaaaacagctataatacctacgcctcagcaatacacagaactgaaaaaacag~g
>Ind.A4
~~~~~~

~~~~~~gattt
caagaaaacagctataatacctacgcctcagcaatacacagaactgaaaaaacag~g
>Ind.A5
~~~~~~gattt
caagaaaacagctataatacctacgcctcagcaatacacagaactgaaaaaacag~g
>Ind.A6
~~~~~~gattt
caagaaaacaactataatacctacgcctcagcaatacacagaactgaaaaaacag~g
>Ind.A7
~~~~~~gattt
caagaaaacagctataatacctacgcctcagcaatacacagaactgaaaaaacag~g
>Ind.A8
~~~~~~gattt
caagaaaacagctataatacctacgcctcagcaatacacagaactgaaaaaacag~g
>Ind.B1
~~~~~~gattt
caagaaaacagctataatacctacgcctcagcaatacacagaactgaaaaaacag~g
>Ind.B2
~~~~~~gattt
caagaaaacagctataatacctacgcctcagcaatacacagaactgaaaaaacag~g
>Ind.B3
~~~~~~gattt
caagaaaacagctataatacctacgcctcagcaatacacagaactgaaaaaacag~g
>Ind.A1
~~~~~~
~~~~~~~~~~~~~~~~~~~~~~~~~~~~~~~~~~~~~~~AGAACTGAAAAAACAG~GACG
TGAGTGGTATGTGGCTCTGAATAAAAGAGGGAAAGCTAAACGAGGCTGCAGCCCACGGGT

>Ind.A2
~~~~~~
~~~~~~~~~~~~~~~~~~~~~~~~~~~~~~~~~~~~~~~AGAACTGAAAAAACAG~GACG
TGAGTGGTATGTGGCTCTGAATAAAAGAGGGAAAGCTAAACGAGGCTGCAGCCCACGGGT

>Ind.A3
~~~~~~
~~~~~~~~~~~~~~~~~~~~~~~~~~~~~~~~~~~~~~~AGAACTGAAAAAACAG~GACG
TGAGTGGTATGTGGCTCTGAATAAAAGAGGGAAAGCTAAATGAGGCTGCAGCCCACGGGT

>Ind.A4
~~~~~~
~~~~~~~~~~~~~~~~~~~~~~~~~~~~~~~~~~~~~~~AGAACTGAAAAAACAG~GACG
TGAGTGGTATGTGGCTCTGAATAAAAGAGGGAAAGCTAAACGAGGCTGCAGCCCACGGGT

>Ind.B1
~~~~~~
~~~~~~~~~~~~~~~~~~~~~~~~~~~~~~~~~~~~~~~AGAACTGAAAAAACAG~GACG

TGAGTGGTATGTGGCTCTGAATAAAAGAGGGAAAGCTAAACGAGGCTGCAGCCCACGGGT

>Ind.B2
~~~~~~
~~~~~~~~~~~~~~~~~~~~~~~~~~~~~~~~~~~~~~~AGAACTGAAAAAACCCTGACG
TGAGTGGTATGTGGCTCTGAATAAAAGAGGGAAAGCTAAACGAGGCTGCAGCCCACAGGT

>Ind.B3
~~~~~~
~~~~~~~~~~~~~~~~~~~~~~~~~~~~~~~~~~~~~~~AGAACTGAAAAAACAG~GACG
TGAGTGGTATGTGGCTCTGAATAAAAGAGGGAAAGCTAAACGAGGCTGCAGCCCACAGGT

>Ind.C1
~~~~~~
~~~~~~~~~~~~~~~~~~~~~~~~~~~~~~~~~~~~~~~agaactgaaaaaacag~gacg
tgagtggtatgtggctctgaataaaagagggaaagctaaacgaggctgcagcccacgggt

>Ind.C2
~~~~~~
~~~~~~~~~~~~~~~~~~~~~~~~~~~~~~~~~~~~~~~agaactgaaaaaacag~gacg
tgagtggtatgtggctctgaataaaagagggaaagctaaacgaggctgcagcccacgggt

>Ind.C3
~~~~~~
~~~~~~~~~~~~~~~~~~~~~~~~~~~~~~~~~~~~~~~agaactgaaaaaacag~gacg
tgagtggtatgtggctctgaataaaagagggaaagctaaacgaggctgcagcccacgggt

>Ind.C4
~~~~~~
~~~~~~~~~~~~~~~~~~~~~~~~~~~~~~~~~~~~~~~agaactgaaaaaacag~gacg
tgagtggtatgtggctctgaataaaagagggaaagctaaacgaggctgcagcccacgggt

>Ind.A1
~~~~~~
~~~~~~
~~~~~~tgcagcccacgggt
caaaccccagcacatctctacccattttttgccgagattcaagcagtcggagcag~~~cc
agaactttcttt
>Ind.A2
~~~~~~
~~~~~~
~~~~~~tgcagcccacgggt
caaaccccagcacatctctacccattttttgccgagattcaagcagtcggagcag~~~cc
agaactttcttt
>Ind.A3
~~~~~~
~~~~~~

~~~~~~
~~~~~~tgcagcccacgggt
caaaccccagcacatctctacccattttttgccgagattcaagcagtcggagcag~~~cc
agaactttcttt
>Ind.A4
~~~~~~
~~~~~~
~~~~~~tgcagcccacgggt
caaaccccagcacatctctacccattttttgccgagattcaagcagtcggagcag~~~cc
agaactttcttt
>Ind.A5
~~~~~~
~~~~~~
~~~~~~tgcagcccacgggt
caaaccccagcacatctctacccattttttgccgaaaagcaagcagtcggagcag~~~cc
agaactttcttt
>Ind.A6
~~~~~~
~~~~~~
~~~~~~tgcagcccacgggt
caaaccccagcacatctctacccattttttgccgagattcaagcagtcggagcag~~~cc
agaactttcttt
>Ind.A7
~~~~~~
~~~~~~
~~~~~~tgcagcccacgggt
caaaccccagcacatctctacccattttttgccgagattcaagcagtcggagcag~~~cc
agaactttcttt
>Ind.A8
~~~~~~
~~~~~~
~~~~~~tgcagcccacgggt
caaaccccagcacatctctacccattttttgccgagattcaagcagtcggagcag~~~cc
agaactttcttt
>Ind.B1
~~~~~~
~~~~~~
~~~~~~tgcagcccacgggt
caaaccccagcacatctctacccattttttgccgagattcaagcagtcggagcag~~~cc
agaactttcttt
>Ind.B2
~~~~~~
~~~~~~
~~~~~~tgcagcccacgggt
caaaccccagcacatctctacccattttttgccgagattcaagcagtcggagcag~~~cc
agaactttcttt
~~~~~~

>Ind.B3
~~~~~~
~~~~~~
~~~~~~tgcagcccacgggt
caaactccagcacatctctacccattttttgccgagattcaagcagtcggggcag~~~cc
agaactttcttt
>Ind.B4
~~~~~~
~~~~~~
~~~~~~tgcagcccacgggt
caaaccccagcacatctctacccattttttgccgagattcaagcagtcggagcag~~~cc
agaactttcttt
>Ind.A1
~~~~~~
~~~~~~
~~~~~~
~~~~~~
agaactttctttcacggtcactg~ttcctgaaaagaaaaagccacctaaccctatcaagc
caaaggttcccctttctgcacct
>Ind.A2
~~~~~~
~~~~~~
~~~~~~
~~~~~~
agaactttctttcacggtcactg~ttcctgaaaagaaaaagccacctaaccctatcaagc
caaaggttcccctttctgcacct
>Ind.A3
~~~~~~
~~~~~~
~~~~~~
~~~~~~
agaactttctttcacggtcactgnttcctgaaaagaaaaagccacctaaccctatcaagc
caaaggttcccctttctgcacct
>Ind.A4
~~~~~~
~~~~~~
~~~~~~
~~~~~~
agaactttctttcacggtcactg~ttcctgaaaagaaaaagccacctaaccctatcaagc
caaaggttccccattctgcacct
>Ind.B1
~~~~~~
~~~~~~
~~~~~~
~~~~~~
agaactttctttcacggtcactg~ttcctgaaaagaaaaagccacctaaccctatcaagc

caaaggttcccctttctgcacct
>Ind.B2
~~~~~~
~~~~~~
~~~~~~
~~~~~~
agaactttctttcacggtcactg~ttcctgaaaagaaaaagccacctaaccctatcaagc
caaaggttcccctttctgcacct
>Ind.B3
~~~~~~
~~~~~~
~~~~~~
~~~~~~
agaactttctttcacggtcactg~ttcctgaaaagaaaaagccacctaaccctatcaagc
caaaggttcccctttctgcacct
>Ind.B4
~~~~~~
~~~~~~
~~~~~~
~~~~~~
agaactttctttcacggtcactg~ttcctgaaaagaaaaagccacctaaccctatcaagc
caaaggttcccctttctgcacct
>Ind.A1
~~~~~~
~~~~~~
~~~~~~
~~~~~~
~~~~~~
~~~~~~ttcccctttctgcacctaggaaaagccctaacacggtgaaatacagactcaagt
ttcgctttggataatattc
>Ind.A2
~~~~~~
~~~~~~
~~~~~~
~~~~~~
~~~~~~
~~~~~~ttcccctttctgcacctaggaaaagccctaacacggtgaaatacagactcaagt
ttcgctttggataatattc
>Ind.A3
~~~~~~
~~~~~~
~~~~~~
~~~~~~
~~~~~~
~~~~~~ttcccctttctgcacctaggaaaagccctaacacggtgaaatacagactcaagt
ttcgctttggataatattc

>Ind.A4
~~~~~~
~~~~~~
~~~~~~
~~~~~~
~~~~~~
~~~~~~ttcccctttctgcacctaggaaaagccctaacacggtgaaatacagactcaagt
ttcgctttggataatattc
>Ind.A5
~~~~~~
~~~~~~
~~~~~~
~~~~~~
~~~~~~
~~~~~~ttcccctttctgcatctaggaaaagccctaacacggtgaaatacagactcaagt
ttcgctttggataatattc
>Ind.A6
~~~~~~
~~~~~~
~~~~~~
~~~~~~
~~~~~~
~~~~~~ttcccctttctgcacctaggaaaagccctaacacggtgaaatacagactcaagt
ttcgctttggataatattc
>Ind.A7
~~~~~~
~~~~~~
~~~~~~
~~~~~~
~~~~~~
~~~~~~ttcccctttctgcacctaggaaaagccctaacacggtgaaatacagactcaagt
ttcgctttggataatattc
>Ind.A8
~~~~~~
~~~~~~
~~~~~~
~~~~~~
~~~~~~
~~~~~~ttcccctttctgcacctaggaaaagccctaacacggtgaaatacagactcaagt
ttcgctttggataatattc
>Ind.B1
~~~~~~
~~~~~~
~~~~~~
~~~~~~
~~~~~~
~~~~~~

~~~~~~ttcccctttctgcacctaggaaaagccctaacacggtgaaatacagactcaagt
ttcgctttggataatattc
>Ind.B2
~~~~~~
~~~~~~
~~~~~~
~~~~~~
~~~~~~
~~~~~~ttcccctttctgcacctaggaaaagccctaacacggtgaaatacagactcaagt
ttcgctttggataatattc
>Ind.B3
~~~~~~
~~~~~~
~~~~~~
~~~~~~
~~~~~~
~~~~~~ttcccctttctgcacctaggaaaagccctaacacggtgaaatacagactcaagt
ttcgctttggataatattc
>Ind.A1
~~~~~~
~~~~~~
~~~~~~
~~~~~~
~~~~~~
~~~~~~ttcccctttctgcacctaggaaaagccctaacacgatgaaatacagactcaagt
ttcgctttggataatattc
>Ind.A2
~~~~~~
~~~~~~
~~~~~~
~~~~~~
~~~~~~
~~~~~~ttcccctttctgcacctaggaaaagccctaacacgatgaaatacagactcaagt
ttcgctttggataatattc
>Ind.A3
~~~~~~
~~~~~~
~~~~~~
~~~~~~
~~~~~~
~~~~~~ttcccctttctgcacctaggaaaagccctaacacgatgaaatacagactcaagt
ttcgctttggataatattc
>Ind.A4
~~~~~~
~~~~~~
~~~~~~

```
~~~~~~~~~~~~~~~~~~~~~~~~~~~~~~~~~~~~~~~~~~~~~~~~~~~~~~~~~~~~
~~~~~~~~~~~~~~~~~~~~~~~~~~~~~~~~~~~~~~~~~~~~~~~~~~~~~~~~~~~~
~~~~~~ttcccctttctgcacctaggaaaagccctaacacggtgaaatacagactcaagt
ttcgctttggataatattc
>Ind.A5
~~~~~~~~~~~~~~~~~~~~~~~~~~~~~~~~~~~~~~~~~~~~~~~~~~~~~~~~~~~~
~~~~~~~~~~~~~~~~~~~~~~~~~~~~~~~~~~~~~~~~~~~~~~~~~~~~~~~~~~~~
~~~~~~~~~~~~~~~~~~~~~~~~~~~~~~~~~~~~~~~~~~~~~~~~~~~~~~~~~~~~
~~~~~~~~~~~~~~~~~~~~~~~~~~~~~~~~~~~~~~~~~~~~~~~~~~~~~~~~~~~~
~~~~~~~~~~~~~~~~~~~~~~~~~~~~~~~~~~~~~~~~~~~~~~~~~~~~~~~~~~~~
~~~~~~ttcccctttctgcacctaggaaaagccctaacacggtgaaatacagactcaagt
ttcgctttggataatattc
>Ind.A6
~~~~~~~~~~~~~~~~~~~~~~~~~~~~~~~~~~~~~~~~~~~~~~~~~~~~~~~~~~~~
~~~~~~~~~~~~~~~~~~~~~~~~~~~~~~~~~~~~~~~~~~~~~~~~~~~~~~~~~~~~
~~~~~~~~~~~~~~~~~~~~~~~~~~~~~~~~~~~~~~~~~~~~~~~~~~~~~~~~~~~~
~~~~~~~~~~~~~~~~~~~~~~~~~~~~~~~~~~~~~~~~~~~~~~~~~~~~~~~~~~~~
~~~~~~~~~~~~~~~~~~~~~~~~~~~~~~~~~~~~~~~~~~~~~~~~~~~~~~~~~~~~
~~~~~~ttcccctttctgcacctaggaaaagccctaacacggtgaaatacagactcaagt
ttcgctttggataatattc
>Ind.A7
~~~~~~~~~~~~~~~~~~~~~~~~~~~~~~~~~~~~~~~~~~~~~~~~~~~~~~~~~~~~
~~~~~~~~~~~~~~~~~~~~~~~~~~~~~~~~~~~~~~~~~~~~~~~~~~~~~~~~~~~~
~~~~~~~~~~~~~~~~~~~~~~~~~~~~~~~~~~~~~~~~~~~~~~~~~~~~~~~~~~~~
~~~~~~~~~~~~~~~~~~~~~~~~~~~~~~~~~~~~~~~~~~~~~~~~~~~~~~~~~~~~
~~~~~~~~~~~~~~~~~~~~~~~~~~~~~~~~~~~~~~~~~~~~~~~~~~~~~~~~~~~~
~~~~~~ttcccctttctgcacctaggaaaagccctaacacggtgaaatacagactcaagt
ttcgctttggataatattc
>Ind.A8
~~~~~~~~~~~~~~~~~~~~~~~~~~~~~~~~~~~~~~~~~~~~~~~~~~~~~~~~~~~~
~~~~~~~~~~~~~~~~~~~~~~~~~~~~~~~~~~~~~~~~~~~~~~~~~~~~~~~~~~~~
~~~~~~~~~~~~~~~~~~~~~~~~~~~~~~~~~~~~~~~~~~~~~~~~~~~~~~~~~~~~
~~~~~~~~~~~~~~~~~~~~~~~~~~~~~~~~~~~~~~~~~~~~~~~~~~~~~~~~~~~~
~~~~~~~~~~~~~~~~~~~~~~~~~~~~~~~~~~~~~~~~~~~~~~~~~~~~~~~~~~~~
~~~~~~ttcccctttctgcacctaggaaaagccctaacacggtgaaatacagactcaagt
ttcgctttggataatattc
```

>Ema10-promoter-5’UTR
GGGGGAGATGTTGTCGAGCACCCATTTCCAAGTTGCAACTCTCCACACCGGCCGCTACCC
GTGGGGGACTCGATCGCTTCCGCTAGGCTGAATTGCGCGATGCGCTGGT~CCTGG~TTGG
GGGTGGCCAGGCAGCGCGTG~AGAACTGCTGGGGTGCGTGG~CCCCGGTGTGCGCGCACC
CAGAGTCTGGGTGCAGCTTCGCTCCGCCAGGTTCTCC~TCCCACTCACTCTCTCCCATCT
CCTCCCTCCTCTAAACACCCCG~CCCCTGGGGCTGCGGGGCTCCCTCTATTTACCGGCCA
G~TGAGTACACAAAGCGGCGGGTG~AGGGG~AAGCTTCGCAGGCGTGCACTAAGCAGTGA
GATCACTGGCGTTATAAATATCCTGGTGCCAGAGCCGAGATCCGCTCGGGTGGCCTCTCT
CTCCCCCTCTTTCT~CCCGAGG~CTATGT~CCACCCGGTGCTGCGAGGGGGGCAGCGCCA
GAGGCACACCGCCGCGCGGGGGCTACAAAGCCCAGGATTAGCCCTGCAA
>Ema6-promoter-5’UTR
GGGGGAGATGTTGTCGAGCACCCATTTCCAAGTTGCAACTCTCCACACCGGCCGCTACCC
GTGGGGGACTCGATCGCTTCCGCTAGGCTGAATTGCGCGATGCGCTGGT~CCTGG~TTGG
GGGTGGCCAGGCAGCGCGTG~AGAACTGCTGGGGTGCGTGG~CCCCGGTGTGCGCGCACC
CAGAGTCTGGGTGCAGCTTCGCTCCGCCAGGTTCTCC~TCCCACTCACTCTCTCCCATCT
CCTCCCTCCTCTAAACACCCCG~CCCCTGGGGCTGCGGGGCTCCCTCTATTTACCGGCCA
G~TGAGTACACAAAGCGGCGGGTG~AGGGG~AAGCTTCGCAGGCGTGCACTAAGCAGTGA
GATCACTGGCGTTATAAATATCCTGGTGCCAGAGCCGAGATCCGCTCGGGTGGCCTCTCT
CTCCCCCTCTTTCT~CCCGAGG~CTATGT~CCACCCGGTGCTGCGAGGGGGGCAGCGCCA
GAGGCACACCGCCGCGCGGGGGCTACAAAGCCCAGGATYAGCCCTGCAA
>KR138-promoter-5’UTR
GGGGAAGATGTTGTCGAGCACCCATTTCCAAGTTGCAACTCTCCACACCCGCCGCTACCC
GTGGGGGACTCGATCGCTTCCGCTAGGCTGAATTGCGCGATGCGCTGGT~CCTGG~TTGG
GGGTGGCCAGGCAGCGCGTG~AGAACTGCTGGGGTGCGTGG~CCCCGGTGTGGGCGCACC
CAGAGTCTGGGTGCAGCTTCGCTCCGCCAGCTTCTCC~TCCCACTCACTCTCTCCCATCT
CCTCCCTCCTCTAAACACCCCG~CCCCTGGGGCTGCGGGGCTCCCTCTATTTACCGGCCA
G~TGAGTACACAAAGCGGCGGGTG~AGGGG~AAGCTTCGCAGGCGTGCACTAAGCAGTGA
GATCACTGGCGTTATAAATATCCTGGTGCCAGAGTCGAGATCCGCTCGGGTGGCCTCTCT
CTCCCCCTCTTTCT~CCCGAGG~CTATGT~CCACCCGGTGCTGCGAGGGGGGCAGCGCCA
GAGGCACACCGCCGCGCGGGGGCTACAAAGCCCAGGATCAGCCCTGCAA
>KR14-promoter-5’UTR
GGGGAAGATGTTGTCGAGCACCCATTTCCAAGTTGCAACTCTCCACACCCGCCGCTACCC
GTGGGGGACTCGATCGCTTCCGCTAGGCTGAATTGCGCGATGCGCTGGT~CCTGG~TTGG
GGGTGGCCAGGCAGCGCGTG~AGAACTGCTGGGGTGCGTGG~CCCCGGTGTGGGCGCACC
CAGAGTCTGGGTGCAGCTTCGCTCCGCCAGCTTCTCC~TCCCACTCACTCTCTCCCATCT
CCTCCCTCCTCTAAACACCCCG~CCCCTGGGGCTGCGGGGCTCCCTCTATTTACCGGCCA
G~TGAGTACACAAAGCGGCGGGTG~AGGGG~AAGCTTCGCAGGCGTGCACTAAGCAGTGA
GATCACTGGCGTTATAAATATCCTGGTGCCAGAGTCGAGATCCGCTCGGGTGGCCTCTCT
CTCCCCCTCTTTCT~CCCGAGG~CTATGT~CCACCCGGTGCTGCGAGGGGGGCAGCGCCA
GAGGCACACCGCCGCGCGGGGGCTACAAAGCCCAGGATCAGCCCTGCAA
>LO3508-promoter-5’UTR
GGGGGAGATGTTGTCGAGCACCCATTTCTAAGTTGCAACTCTCCACACCCGCCGCTACCC
GTGGGGGACTCGATCGCTTCCGCTAGGCTGAATTGCGCGATGCGCTGGT~CCTGG~TTGG
GGGTGGCCAGGCAGCGCGTG~AGAACTGCTGGGGTGCGTGG~CCCCGGTGTGGGCGCACC
CAGAGTCTGGGTGCAGCTTCGCTCCGCCAGCTTCTCC~TCCCACTCACTCTCTCCCATCT
CCTCCCTCCTCTAAACACCCCG~CCCCTGGGGCTGCGGGGCTCCCTCTATTTACCGGCCA
G~TGAGTACACAAAGCGGCGGGTG~AGGGG~AAGCTTCGCAGGCGTGCACTAAGCAGTGA
GATCACTGGCGTTATAAATATCCTGGTGCCAGAGCCGAGATCCGCTGGGGTGGCCTCTCT

CTCCCCCTCTTTCT~CCCGAGG~CTATGT~CCACCCGGTGCTGCGAGGGGGGCAGCGCCA
GAGGCACACCGCCGCGCGGGGGCTACAAAGCCCAGGATCAGCCCTGCAA
>LO3505-promoter-5’UTR
GGGGGAGATGTTGTCGAGCACCCATTTCTAAGTTGCAACTCTCCACACCCGCCTCTACCC
GTGGGGGACTCGATCGCTTCCGCTAGGCTGAATTGCGCGATGCGCTGGTCCTGGTTGGGG
GTGGCCAGGCAGCGCGTGAGAACTGCTGGGGTGCGTGGCCCCGGTGTGGGCGCACCCAGA
GTCTGGGTGCAGCTTCGCTCCGCCAGCTTCTCCTCCCACTCACTCTCTCCCATCTCCTCC
CTCCTCTAAACACCCCGCCCCTGGGGCTGCGGGGCTCCCTCTATTTACCGGCCAGTGAGT
ACACAAAGCGGCGGGTG A/Δ
GGGGAAGCTTCGCAGGCGTGCACTAAGCAGTGAGATCACTGGCGTTATAAATATCCTGGT
GCCAGAGCCGAGATCCGCTGGGGTGGCCTCTCTCTCCCCCTCTTTCTCCCGAGGCTATGT
CCACCCGGTGCTGCGAGGGGGGCAGCGCCAGAGGCACACCGCCGCGCGGGGGCTACAAAG
CCCAGGATCAGCCCTGCAA
>Ind.A.1
~~~~~~~~~~~~~~~~~~~~~~~~~~~~CAAGTTGCAACTCTCCACACCCGCCGCTACCC
GTGGGGGACTCGATCGCTTCCGCTAGGCTGAATTGCGCGATGCGCTGGT~CCTGG~TTGG
GGG~~~~~~~~~~
>Ind.A.2
~~~~~~~~~~~~~~~~~~~~~~~~~~~~CAAGTTGCAACTCTCCACACCCGCCGCTACCC
GTGGGGGACTCGATCGCTTCCGCTAGGCTGAATTGCGCGATGCGCTGGT~CCTGG~TTGG
GGG~~~~~~~~~~
>Ind.A.3
~~~~~~~~~~~~~~~~~~~~~~~~~~~~CAAGTTGCAACTCTCCACACCCGCCGCTACCC
GTGGGGGACTCGATCGCTTCCGCTAGGCTGAATTGCGCGATGCGCTGGT~CCTGG~TTGG
GGG~~~~~~~~~~
>Ind.A.4
~~~~~~~~~~~~~~~~~~~~~~~~~~~~CAAGTTGCAACTCTCCACACCCGCCGCTACCC
GTGGGGGACTCGATCGCTTCCGNTAGGCTGAATTGCGCGATGCGCTGGTTCCTGG~TTGG
GGG~~~~~~~~~~
>Ind.A.5
~~~~~~~~~~~~~~~~~~~~~~~~~~~~CAAGTTGCAACTCTCCACACCCGCCGCTACCC
GTGGGGGACTCGATCGCTTCCGCTAGGCTGAATTGCGCGATGCGCTGGT~CCTGG~TTGG
GGG~~~~~~~~~~
>Ind.B.1
~~~~~~~~~~~~~~~~~~~~~~~~~~~~CAAGTTGCAACTCTCCACACCCGCCGCTACCC
GTGGGGGACTCGATCGCTTCCGCTAGGCTGAATTGCGCGATGCGCTGGT~CCTGG~TTGG
GGG~~~~~~~~~
>Ind.B.2
~~~~~~~~~~~~~~~~~~~~~~~~~~~~CAAGTTGCAACTCTCCACACCCGCCGCTACCC
GTGGGGGACTCGATCGCTTCCGCTAGGCTGAATTGCGCGATGCGCTGGT~CCTGG~TTGG
GGG~~~~~~~~~
>Ind.B.3
~~~~~~~~~~~~~~~~~~~~~~~~~~~~CAAGTTGCAACTCTCCACACCCGCCGCTACCC
GTGGGGGACTCGATCGCTTCCGCTAGGCTGAATTGCGCGATGCGCTGGT~CCTGG~TTGG
GGG~~~~~~~~~
>Ind.B.4
~~~~~~~~~~~~~~~~~~~~~~~~~~~~CAAGTTGCAACTCTCCACACCCGCCGCTACCC
GTGGGGGACTCGATCGCTTCCGCTAGGCTGAATTGCGCGATGCGCTGGT~CCTGG~TTGG
~~~~~~~~~~~~~~~~~~~~~~~~~~~~

GGG~~~~~~~~~
>Ind.B.5
~~~~~~~~~~~~~~~~~~~~~~~~~~~~CAAGTTGCAACTCTCCACACCCGCCGCTACCC
GTGGGGGACTCGATCGCTTCCGNTAGGCTGAATTGCGCGATGCGCTGGT~CCTGG~TTGG
GGG~~~~~~~~~
>Ind.A.1
~~~~~~
~~~~~~CTGG~TTGG
GGGTGGCCAGGCAGCGCGTG~AGAACTGCTGGGGTGCGTGG~CCCCGGTGTGGGCGCACC
CAGAGTCTGGGTGCAGCTTCGCTCCGCCAGCT~~~~~~~
>Ind.A.2
~~~~~~
~~~~~~CTGG~TTGG
GGGTGGCCAGGCAGCGCGTG~AGAACTGCTNNGGTGCGTGG~CCCCGGTGTGGGCGCACC
CAGAGTCTGGGTGCAGCTTCGCTCCGCCAGCT~~~~~~~
>Ind.B.1
~~~~~~
~~~~~~CTAG~TTGG
GGGTAGCCAGGCAGCGCGTG~AGAACTGCTGGGGTGCGTGG~CCCCGGTGTGGGCACACC
CAGAGTCTGGGTGCAGCTTCGCTCCGCCAGCT~~~~~~~
>Ind.B.2
~~~~~~
~~~~~~CTAG~TTGG
GGGTAGCCAGGCAGCACGTG~AGAACTGCTGGGGTGCGTGG~CCCCGGTGTGGGCACACC
CAGAGTCTGGGTGCAGCTTCGCTCCGCCAGCT~~~~~~~
>Ind.B.3
~~~~~~
~~~~~~CTGG~TTGG
GGGTGGCCAGGCAGCGCGTG~AGAACTGCTGGGGTGCGTGG~CCCCGGTGTGGGCGCACC
CAGAGTCTGGGTGCAGCTTCGCTCCGCCAGCT~~~~~~~
>Ind.B.4
~~~~~~
~~~~~~CTGG~TTGG
GGGTGGCCAGGCAGCGCGTG~AGAACTGCTGGGGTGCGTGG~CCCCGGTGTGGGCGCACC
CAGAGTCTGGGTGCAGCTTCGCTCCGCCAGCT~~~~~~~
>Ind.B.5
~~~~~~
~~~~~~CTGG~TTGG
GGGTGGCCAGGCAGCGCGTG~AGAACTGCTGAGGTGCGTGG~CCCCGGTGTGGGCGCACC
CAGAGTCTGGGTGCAGCTTCGCTCCGCCAGCT~~~~~~~
>Ind.B.6
~~~~~~
~~~~~~CTGGCTTGG
GGGTGGCCAGGCAGCGCNTGCAGAACTGCTGGGGTGCNTGGAGCCCGGTGTGGGCGCACC
CAGAGTCTGGGTGCAGCTTCGCTCCGCCAGCT~~~~~~~
>Ind.A.1
~~~~~~
~~~~~~
~~~~~~

~~~~~~
~~~~~~~~~~~~~~~~~~~~~~~~~~~~~GCTTCTCC~TCCCACTCACTCTTTCCCATCT
CCTCCCTTCTCTAAACANGAAG~NCCCTGGGGCTGCGGGGCTCCCTCTATTTACCGGC~~
~~~~
>Ind.A.2
~~~~~~~~~~~~~~~~~~~~~~~~~~~~~~~~~~~~~~~~~~~~~~~~~~~~~~~~~~~~
~~~~~~~~~~~~~~~~~~~~~~~~~~~~~~~~~~~~~~~~~~~~~~~~~~~~~~~~~~~~
~~~~~~~~~~~~~~~~~~~~~~~~~~~~~~~~~~~~~~~~~~~~~~~~~~~~~~~~~~~~
~~~~~~~~~~~~~~~~~~~~~~~~~~~~~GCTTCTCC~TCCCACTCACTCTCTCCCATCT
CCTCCCTTCTCTAAACACCCCG~CCCCTGGGGCTGCGGGGCTCCCTCTATTTACCGGC~~
~~~~
>Ind.A.3
~~~~~~
~~~~~~
~~~~~~
~~~~~~~~~~~~~~~~~~~~~~~~~~~~~GCTTCTCC~TCCCACTCACTCTCTCCCATCT
CCTCCCTTCTTTAAACACCCCGACCCCTGGGGCTGCGGGGCTCCCTCTATTTACCGGC~~
~~~~
>Ind.A.4
~~~~~~~~~~~~~~~~~~~~~~~~~~~~~~~~~~~~~~~~~~~~~~~~~~~~~~~~~~~~
~~~~~~~~~~~~~~~~~~~~~~~~~~~~~~~~~~~~~~~~~~~~~~~~~~~~~~~~~~~~
~~~~~~~~~~~~~~~~~~~~~~~~~~~~~~~~~~~~~~~~~~~~~~~~~~~~~~~~~~~~
~~~~~~~~~~~~~~~~~~~~~~~~~~~~~GCTTCTCC~TCCCACTCACTCTCTCCCATCT
CCTCCCTTCTCTAAACACCCCG~CCCCTGGGGCTGCGGGGCTCCCTCTATTTACCGGC~~
~~~~
>Ind.A.5
~~~~~~
~~~~~~
~~~~~~
~~~~~~~~~~~~~~~~~~~~~~~~~~~~~GCTTCTCC~TCCCACTCACTCTCTCCCATCT
CCTCCCTTCTCTAAACACCCCG~CCCCTGGGGCTGCGGGGCTCCCTCTATTTACCGGC~~
~~~~
>Ind.B.1
~~~~~~~~~~~~~~~~~~~~~~~~~~~~~~~~~~~~~~~~~~~~~~~~~~~~~~~~~~~~
~~~~~~~~~~~~~~~~~~~~~~~~~~~~~~~~~~~~~~~~~~~~~~~~~~~~~~~~~~~~
~~~~~~~~~~~~~~~~~~~~~~~~~~~~~~~~~~~~~~~~~~~~~~~~~~~~~~~~~~~~
~~~~~~~~~~~~~~~~~~~~~~~~~~~~~GCTTCTCC~TCCCACTCACTCTCTCCCATCT
CCTCCCTTCTCTAAACACCCNG~CCCCTGGGGCTGCGGGGCTCCCTCTATTTACCGGC~~
~~~~
>Ind.B.2
~~~~~~
~~~~~~
~~~~~~
~~~~~~~~~~~~~~~~~~~~~~~~~~~~~GCTTCTCC~TCCCACTCACTCTCTCCCATCT
CCTCCCTTCTCTAAACACNNNN~CCCCTGGGGCTGCGGGGCTCCCTNTATTTACCGGC~~
~~~~
>Ind.B.3
~~~~~~~~~~~~~~~~~~~~~~~~~~~~~~~~~~~~~~~~~~~~~~~~~~~~~~~~~~~~

```
~~~~~~~~~~~~~~~~~~~~~~~~~~~~~~~~~~~~~~~~~~~~~~~~~~~~~~~~~~~~
~~~~~~~~~~~~~~~~~~~~~~~~~~~~~~~~~~~~~~~~~~~~~~~~~~~~~~~~~~~~
~~~~~~~~~~~~~~~~~~~~~~~~~~~~~GCTTCTCC~TCCCACTCACTCTCTCCCATTT
CCTCCCTTCTCTAAACACCCCGACCCCTGGGGCTGCGGGGCTCCCTCTATTTACCGGC~~
~~~~
>Ind.B.4
~~~~~~
~~~~~~
~~~~~~
~~~~~~~~~~~~~~~~~~~~~~~~~~~~~GCTTCTCC~TCCCACTCACTCTCTCCCATCT
CCTCCCTTCCCTAAACACCGGNACCCCTGGGGCTGCGGGGCTCCCTCTATTTACCGGC~~
~~~~
>Ind.B.5
~~~~~~~~~~~~~~~~~~~~~~~~~~~~~~~~~~~~~~~~~~~~~~~~~~~~~~~~~~~~
~~~~~~~~~~~~~~~~~~~~~~~~~~~~~~~~~~~~~~~~~~~~~~~~~~~~~~~~~~~~
~~~~~~~~~~~~~~~~~~~~~~~~~~~~~~~~~~~~~~~~~~~~~~~~~~~~~~~~~~~~
~~~~~~~~~~~~~~~~~~~~~~~~~~~~~GCTTCTCCCACCCACTCACTCTCTCCCATCT
CCTCCCTTCTCTAAACACCCCGACCCCTGGGGCTGCGGGGCTCCCTCTATTTACCGGC~~
~~~~
>Ind.A.1
~~~~~~
~~~~~~
~~~~~~
~~~~~~
~~~~~~GCCA
G~TGAGTACACAAAGCGGCGGGTG~AGGGG~AAGCTTCGCAGGCGTGCACTAAGCAGTGA
GATCACTGGCGTTATA~~~
>Ind.A.2
~~~~~~
~~~~~~
~~~~~~
~~~~~~
~~~~~~GCCA
G~TGAGTACACAAAGCGGCGGGTG~AGGGG~AAGCTTCGCAGGCGTGCACTAAGCAGTGA
GATCACTGGCGTTATA~~~
>Ind.A.3
~~~~~~
~~~~~~
~~~~~~
~~~~~~
~~~~~~GCCA
A~TGAGTACACAAAGCGGCGGGTG~AGGGG~AAGCTTCGCAGGCGTGCACTAAGCAGTGA
GATCACTGGCGTTATA~~~
>Ind.A.4
~~~~~~
~~~~~~
~~~~~~
~~~~~~
```

~~~~~~GCCA
G~TGAGTACACAAAGCGGCGGGTGNAGGGG~AAGCTTCGTAGGCGTGCACTAAGCAGTGA
GATCATTGGCGTTATA~~~
>Ind.A.5
~~~~~~
~~~~~~
~~~~~~
~~~~~~
~~~~~~GCCA
G~TGAGTACACAAAGCGGCGGGTG~AGGGG~AAGCTTCGCAGGCGCGCACTAAGCAGTGA
GATCATTGGCGTTATA~~~
>Ind.B.1
~~~~~~
~~~~~~
~~~~~~
~~~~~~
~~~~~~GCCA
G~TGAGTACACAAAGCGGCGGGTG~AGGGG~AAGCTTCGCAGGCGTGCACTAAGCAGTGA
GATCACTGGCGTTATA~~~
>Ind.B.2
~~~~~~
~~~~~~
~~~~~~
~~~~~~
~~~~~~GCCA
GNTGAGTACACAAAGCGGCGGGTGNAGGGGNAAGCTTCGCAGGCGTGCACTAAGCAGTGA
GATCACTGGCGTTATA~~~
>Ind.B.3
~~~~~~
~~~~~~
~~~~~~
~~~~~~
~~~~~~GCCA
G~TGAGTACATAAAGCGGCGGGTG~AGGGG~AAGCTTCNCAGGCGTGCACTAAGCAGTGA
GATCACTGGCGTTATA~~~
>Ind.B.4
~~~~~~
~~~~~~
~~~~~~
~~~~~~
~~~~~~GCCA
G~TGAGTACACAAAGCGGCGGGTG~AGGGG~AAGCTTCGCAGGCGTGCACTAAGCAGTGA
GATCACTGGCGTTATA~~~
>Ind.B.5
~~~~~~
~~~~~~
~~~~~~
~~~~~~

~~~~~~GCCA
G~TGAGTACACAAAGCGGCGGGTG~AGGGG~AAGCTTCNCAGGCGTGCACTAAGCAGTGA
GATCACTGGCGTTATA~~~
>Ind.A.1
~~~~~~
~~~~~~
~~~~~~
~~~~~~
~~~~~~
~~~~~~
~~~~~~~~~~~TTATAAATATCCTGGTGCCAGAGCCGAGATCCGCTCGGGTGGCCTCTCT
CTCCCCCTCTTNNTACCCGAGGNCCATGT~CCACCCGGTGCTGCGAGGGGGG
>Ind.A.2
~~~~~~
~~~~~~
~~~~~~
~~~~~~
~~~~~~
~~~~~~
~~~~~~~~~~~TTATAAATATCCTGGTGCCAGAGCCGAGATCCGCTCGGGTGGCCTCTCT
CTCCCCCTCTTTCT~CCCGAGG~CTATGT~CCACCCGGTGCTGCGAGGGGGG
>Ind.A.3
~~~~~~
~~~~~~
~~~~~~
~~~~~~
~~~~~~
~~~~~~
~~~~~~~~~~~TTATAAATATCCTGGTGCTAGAGCCGAGATCCGCTCNGGTGGCCTCTCT
CTCCCCCTCTTTCT~CCCGAGG~CTATGT~CCACCCGGTGCTGCGAGGGGGG
>Ind.A.4
~~~~~~
~~~~~~
~~~~~~
~~~~~~
~~~~~~
~~~~~~
~~~~~~~~~~~TTATAAATATCCTGGTGCCAGAGCCGAGATCCGCTCGGGTGGCCTCTCT
CTCCCCCTCTTTCTNCCCGAGG~CTATGT~CTACCCGGTGCTGCGAGGGGGG
>Ind.B.1
~~~~~~
~~~~~~
~~~~~~
~~~~~~
~~~~~~
~~~~~~
~~~~~~~~~~~TTATAAATATCCTGGTGCCAGAGCCGAGATCCGCTCGGGTGGCCTCTCT
CTCCCCCTCTTTCT~CCCGAGG~CTATGT~CCACCCGGTGCTGCGAGGGGGG

>Ind.B.2
~~~~~~
~~~~~~
~~~~~~
~~~~~~
~~~~~~
~~~~~~
~~~~~~~~~~~TTATAAATATCCTGGTGCCAGAGCCGAGATCCGCTCGGGTGGCCTCTCT
CTCCCCCTCTTTCT~CCCGAGG~CTGTGT~CCACCCGGTGCTGCGAGGGGGG
>Ind.B.3
~~~~~~
~~~~~~
~~~~~~
~~~~~~
~~~~~~
~~~~~~
~~~~~~~~~~~TTATAAATATCCTGGTGCCAGAGCCGAGATCCGCTCGGGTGGCCTCTCT
CTCCCCCTCTTTCTNCCCGAGG~CTATGTNCCACCCGGTGCTGCGAGGGGGG
>Ind.B.4
~~~~~~
~~~~~~
~~~~~~
~~~~~~
~~~~~~
~~~~~~
~~~~~~~~~~~TTATAAATATCCTGGTGCCAGAGCCGAGATCCGCTCGGGTGGCCTCTCT
CTCCCCCTCTTTCTNCCCGAGG~CTATGT~CCACCCGGTGCTGCGAGGGGGG
>Ind.B.5
~~~~~~
~~~~~~
~~~~~~
~~~~~~
~~~~~~
~~~~~~
~~~~~~~~~~~TTATAAATATCCTGGTGCCAGAGCCGAGATCCGCTCGGGTGGCCTCTCT
CTCCCCCTCTTTCTACCCGAGG~CTATGT~CCACCCGGTGCTGCGAGGGGGG
